# Attachment, grandiose and vulnerable narcissism, interpersonal competence, and problematic social media use: A latent profile analysis

**DOI:** 10.1371/journal.pone.0359569

**Published:** 2026-10-21

**Authors:** Chris A. Bjornsen, Allen G. Harbaugh

**Affiliations:** 1 Department of Psychology, Longwood University, Farmville, Virginia, United States of America; 2 Department of Mathematics and Computer Science, Longwood University, Farmville, Virginia, United States of America; University of Glasgow, UNITED KINGDOM OF GREAT BRITAIN AND NORTHERN IRELAND

## Abstract

In a cross-sectional study we examined associations between attachment anxiety and avoidance, grandiose narcissism, vulnerable narcissism, interpersonal competence, and problematic social media use (PSMU) in order to enhance the understanding of the characteristics that may contribute to PSMU. Participants (*N* = 555) were recruited from social media and crowdsourcing apps and completed a self-report online questionnaire. Correlations between variables generally supported previous findings, with the exceptions of the negative association between grandiose narcissism and attachment avoidance, and the non-significant association between grandiose narcissism and PSMU. Latent profile analysis (LPA) produced four separate profiles distinguished by levels of attachment anxiety and avoidance characteristic of the secure, avoidant, anxious, and fearful attachment styles. The four profiles were also distinguished by unique combinations of grandiose narcissism, vulnerable narcissism, and interpersonal competence, and suggest differences between the anxious and fearful attachment styles that have been overlooked in previous studies. We validated the LPA results by comparing mean PSMU scores among the four profiles, revealing statistically significant differences between all four profiles. Securely attached participants reported the lowest level of PSMU, with increasing levels of PSMU reported by avoidant, anxious, and fearful participants.

## Introduction

In the present study we examine the associations between attachment anxiety, attachment avoidance, grandiose narcissism (GN), vulnerable narcissism (VN), interpersonal competence (IC), and problematic social media use (PSMU). The primary objective of the study is to investigate patterns of associations between attachment anxiety, attachment avoidance, GN, VN, and IC, and how such patterns may predict levels of PSMU. As there are no extant studies examining these variables together in one study, our introduction below reviews the current literature on relations between the above variables. We hypothesize and test bivariate relations between the above variables and, while these variable-centered analyses are intended to add to the existing literature regarding associations between attachment styles, GN, VN, and IC, our primary objective is to provide context and support for the central predictions of the current study, namely that a person-centered analytic approach (latent profile analysis [LPA]) will identify subgroups within the sample that correspond to the secure, avoidant, anxious, and fearful attachment styles characterized by predicted levels of attachment anxiety, attachment avoidance, GN, VN, and IC, and the identified subgroups or profiles will be distinguished by different levels of PSMU in an auxiliary analysis.

### Attachment style and narcissism

The inherent tendency to create and maintain intimate emotional bonds with specific others throughout life is a basic component of human nature [1], and is associated with psychological and interpersonal competence, well-being, resilience, and emotional stability [2]. Attachment style was historically conceived as a categorical construct encompassing secure, anxious/ambivalent, avoidant/dismissing, and fearful/disorganized types [3–5]. A secure attachment style is characterized by a high degree of trust in close relationships, comfort with closeness and interdependence, and constructive coping in response to stress. The anxious attachment style is characterized by a strong desire for closeness and protection, intense worry about the availability of significant others, and strategies designed to maintain contact with and solicit attention from others. The avoidant attachment style is characterized by discomfort with closeness and dependence on significant others, a preference for emotional distance, and suppression of a desire to seek comfort from others when experiencing distress. Lastly, the fearful attachment style is characterized by a strong internal desire for relationship closeness combined with a high level of fear of the possible negative consequences of reliance on others. Fearful individuals thus tend to demonstrate unusual fluctuations between anxiety and avoidance in response to distress [2]. More recent evidence suggests a two-dimensional model of attachment that addresses levels of anxiety and avoidance as separate continuous variables constitutes an improved conceptualization of attachment representations in adulthood [6–9] and can additionally facilitate a reliable and valid categorical-level perspective when the anxiety and avoidant dimensions are assessed together [10]. Low levels of attachment anxiety and avoidance, analogous to secure attachment, are associated with more positive psychological and interpersonal well-being [8,11]. Conversely, a higher level of attachment anxiety promotes a defense against anticipated loss of relationship satisfaction through constant attention-seeking, while a higher level of attachment avoidance is reflected in greater emotional or interpersonal distance from others who are viewed as unsupportive [12].

Attachment anxiety and avoidance may promote different strategies for managing narcissistic insecurities [13]. Although GN and VN may overlap to a degree [14] and may reflect relatively distinct dimensions on a narcissism spectrum [15], there is mixed evidence regarding the correlation between GN and VN, with some studies indicating a lack of association [16,17] and other research indicating a weak positive association between the two [15]. According to the narcissism spectrum model, the “majority of people with high grandiosity will not be especially high on vulnerability (and vice versa)” [15 p. 20]. Individuals with narcissistic tendencies typically fit one of the two types more strongly, with different underlying emotional self-concepts that may have different etiologies [18] which manifest as distinct behavioral tendencies [19–21]. Overlap between the two types may be accounted for by self-centeredness, entitlement, arrogance [15] and/or antagonism [19,20,22]. GN is characterized by high extraversion, a heightened need for admiration combined with a lack of empathy and a tendency to enhance one’s self-concept by manipulating others or engaging in antisocial behavior. VN is also characterized by an elevated need for admiration from others, but is combined with a tendency toward shyness, a dominant sense of shame regarding the strong need for others, a hypersensitivity to imagined criticism from others, and failures of self-enhancement [14]. The difference between GN and VN is also described as reflecting narcissistic boldness (an approach strategy) vs narcissistic reactivity (an avoidance strategy) [15]. While much of the theoretical and clinical literature on narcissism focuses on pathological levels of this trait, it is argued that “even relatively healthy people can develop narcissistic symptoms after experiencing more or less severe narcissistic threats or humiliation” [23 p. 114], which aligns with the widespread use of measures such as the Short Dark Triad [24] and the Dirty Dozen [25], instruments designed to measure sub-clinical levels of narcissism [26–28].

Supporting clinical and theoretical arguments [13,29,30], GN is often associated with avoidant attachment, while VN is more commonly related to anxious attachment [30–34]. However, some research indicates that VN predicts anxiety and avoidance and is more strongly predictive of avoidance than is GN [19,35]. Relations between attachment and GN are less consistent; some research indicates it is associated with neither anxious nor avoidant attachment [19,21], other research indicates GN is positively related to avoidant attachment [33,35], while another recent study indicates GN is negatively associated with anxious attachment [36]. While comparatively little research exists examining the fearful style compared to secure, anxious, and avoidant styles [37], the fearful attachment style is associated with interpersonal difficulty [38], especially in those with high VN [39].

### Attachment and interpersonal competence

Studies show that avoidant attachment is negatively associated with social and emotional expressivity [40–42], interpersonal communication competence [43], emotional competence, communication, and engagement with others [44], ability to initiate relationships and give emotional support [45], emotional and social expressivity, sensitivity, and control [46], overall interpersonal competence [47], motivation for social contact [48], and relying on others to regulate one’s emotions [49]. Attachment avoidance is also associated with being more interpersonally domineering, vindictive, and cold [50].

Anxious attachment is negatively associated with interpersonal communication competence [43], ability to initiate relationships and give emotional support [45], engagement with others [44], emotional and social expressivity [46], and conciliatory relationship behaviors [51]. Anxious attachment is also positively associated with negative affect, stress, perceived social rejection [48], social sensitivity [40,46], relying on others to regulate one’s emotions [49], and being more interpersonally passive, exploitable, overly nurturant, and intrusive [50]. However, other research indicates anxious attachment is associated with a higher level of relationship-focused disclosure [41, 42]. Combining the avoidant and anxious dimensions, fearful attachment is associated with lower levels of perceived social support [39], social and emotional expressivity [40], relationship commitment [38], physical and emotional closeness, behaviors that benefit oneself or one’s partner/relationship, and conciliatory relationship behaviors [51], as well as higher levels of emotion dysregulation [39] and social sensitivity [40].

### Attachment and social media use

Both systematic reviews [52–54] and a meta-analysis [55] regarding relations between attachment styles and social media (SM) use indicate that high levels of use, and addictive or PSMU, are more strongly and consistently positively associated with anxious attachment than with avoidant attachment. Subsequent studies also report that anxious (but not avoidant) attachment is positively related to PSMU [56,57]. Further, person-centered data analysis strategies such as LPA and latent class analysis (LCA) demonstrate the reliability of using the two-dimensional Experiences in Close Relationships scale (ECR) to classify individuals into groups that correspond to the secure, avoidant, anxious, and fearful attachment styles [10]. Results of other person-centered studies concur with the relations between attachment styles and PSMU reported in more traditional variable-centered research. In one study, a high risk of addiction to social media group demonstrated high levels of SM use, social media addiction (SMA), and anxious attachment [58]. A second study found that both anxious and avoidant attachment dimensions are more strongly endorsed by participants clustered in a problematic smartphone use group (compared to other groups labeled connected or distracted), although attachment anxiety more directly predicted problematic use [59].

Extant studies most commonly report low-to-moderate positive associations between fearful attachment and problematic smartphone use [56,60–63]. However, other studies find no association between a fearful attachment style and problematic smartphone use or SMA [64,65]. Thus, similar to the relations between attachment and narcissism, the relation between anxious attachment and PSMU appears fairly consistent, while the associations between avoidant and fearful attachment styles and PSMU are less consistent.

### Narcissism and interpersonal competence

GN is generally associated with more positive short-term than long-term interpersonal relationship functioning [66–68], including friendship appreciation and intimacy [69] perhaps in part due to the generally higher levels of interpersonal distrust associated with GN [70]. People demonstrating higher VN tend to report greater interpersonal distress than people with higher GN [32], perhaps given the tendency for those with high GN toward positive self-esteem and independent self-construal, compared to a tendency for those high on VN toward low self-esteem and a self-construal of interdependence on others [34]. High VN also tends to be associated with a higher level of emotion dysregulation [35,71] which is associated with lower levels of IC [72].

### Narcissism and social media use

Early theory, measures, and research concerning relations between narcissism and PSMU focus on GN, while research on VN is more recent [14,16]. In meta-analyses addressing primarily GN, higher levels of narcissism predict higher levels of SM use, greater number of SM friends, and greater frequency of SM activity and posting (including photos), reflecting the GN goals of self-enhancement, attention-seeking, and gaining admiration from others [73–75]. A separate review of the literature reported consistent positive associations between both GN and VN and problematic Facebook use, although associations with different SM apps or platforms are less consistent [76]. More recent studies support that GN is positively associated with addictive SM use [77] and time spent using Twitter, Facebook, and Instagram [78]. Further, multiple studies indicate that VN is a stronger predictor of PSMU than GN [79–81]. VN is positively associated with various aspects of PSMU, motivated (in contrast to GN) by a desire to increase positive feelings, decrease negative feelings, and avoid social rejection [82]. Moreover, evidence indicates that VN, as measured by the Hypersensitive Narcissism Scale (HNS) [16], contains two facets: a hypersensitivity factor (which is associated with more time using SM per day, more frequent posting to SM, more concern about obtaining likes and comments, greater jealousy and social comparison to others, greater concern about negative remarks, and greater difference in how the self is portrayed online vs in real life), and an egocentricity factor (which is associated with caring less about SM ‘likes,’ lower jealousy of others, and less concerns about negative remarks from others) [83,84]. Hypersensitivity may drive a high level of SM use, which serves as a context in which vulnerable narcissists can control self-presentations, avoid the anxiety of real-life social interactions [83], and exert greater control over their privacy [85].

### Interpersonal competence and social media use

Various studies report negative associations between SM use or PSMU and IC in adults. The amount of time spent on Facebook is inversely related to ability to initiate relationships [45], and Facebook addiction is negatively associated with communication, conflict resolution, and overall interpersonal skills [86]. Further, a higher level of narcissistic self-presentation on Facebook is associated with a lower level of face-to-face acceptance by peers [87]. Studies reveal that amount of time spent using various online media is negatively associated with IC [88], and higher smartphone addiction is inversely related to the ability to manage interpersonal conflict [89], healthy interpersonal relationships [90], and interpersonal communication [91].

Our review of the literature regarding the relations between the constructs examined in our study suggest a number of predictions related to the primary objective of our study, namely the investigation of patterns of associations between attachment styles, GN, VN, and IC, and how those patterns may predict levels of PSMU. As a reference, we provide a summary in Table 1 below of the variables/constructs in our study.

**Table 1:** Summary descriptions of constructs and definitions.

Construct	Description	Measure (Sample item)
Attachment anxiety	High anxiety reflects a strong desire for close relationships, intense worry about partner availability and value to one’s partner	Experiences in Close Relationships – 12 [8], six items. (“I worry about being abandoned.”)
Attachment avoidance	High avoidance reflects discomfort with closeness and dependence on others, a preference for emotional distance	Experiences in Close Relationships – 12 [8], six items. (“I don’t feel comfortable opening up to romantic partners.”)
Grandiose narcissism (GN)	High GN reflects the perception of one’s self-importance and entitlement, superior qualities compared to others, a general lack of empathy toward others, and manipulation of others for one’s personal benefit.	Narcissism scale of the Short Dark Triad [24], nine items. (“People see me as a natural leader.”)
Vulnerable narcissism (VN)	High VN reflects an excessive need for admiration from others, a tendency to experience shame regarding one’s need for others, hypersensitivity to imagined or real criticism from others, and inability to overtly elicit desired attention from others	Hypersensitive Narcissism Scale (HNS) [16], ten items. (“My feelings are easily hurt by ridicule or the slighting remarks of others.”)
Interpersonal competence (IC)	High IC reflects perceiving oneself as capable of initiating relationship interactions, assertively defending oneself from negative interpersonal intrusions, appropriate self-disclosure of sensitive, personal information, ability to emotionally support others, and ability to manage conflict in relationships.	Interpersonal Competence Questionnaire (ICQ-15) [93], fifteen items. (“Indicate your level of competence and comfort handling each of the following: Letting a new companion get to know the “real” you.”)
Problematic social media use (PSMU)	High PSMU reflects a tendency to be controlled by rather than to exert agentic control of social media. PSMU is characterized by impulsive, compulsive use, use designed to elevate one’s mood, and using social media despite negative consequences.	Original scale, six items. (“I often check my social media impulsively, for no reason.”)

### Hypotheses

Following the evidence discussed above, in the current study we predict the following variable-centered (H1 - H7) and person-centered (H8, H9) outcomes. H1: Attachment anxiety is positively associated with VN. H2: Attachment avoidance is positively associated with GN. H3: Both attachment avoidance and anxiety are negatively associated with IC. H4: Attachment anxiety and avoidance are positively associated with PSMU, with a stronger association between anxiety and PSMU. H5: VN is negatively associated with IC. H6: Both GN and VN are positively associated with PSMU, with a stronger association between VN and PSMU. H7: IC and PSMU are negatively associated. H8: A person-centered analysis (LPA) will reveal profiles reflecting the four traditional attachment styles and associations between avoidance, anxiety, GN, VN, and IC predicted in H1 - H3 and H5. The prediction that separate profiles exist distinguished primarily by attachment anxiety and avoidance is based on the developmental progression of the constructs or characteristics measured in the current study. In a general developmental sequence, attachment originates in infancy and toddlerhood, and high anxiety and/or avoidance would then drive the potential development of narcissism [13,29]. Together, attachment anxiety and avoidance and narcissism (or lack thereof), should therefore underlie the development of levels of IC and, finally, SM use. H9: Finally, we predict that, should H8 be supported, an auxiliary analysis (used to validate the profiles [92]) will demonstrate that the profile groups will differ on levels of PSMU, with a secure profile reporting the lowest level of PSMU and the insecure profile groups (avoidant, anxious, and fearful) reporting higher levels of PSMU.

## Materials and methods

### Procedure and participants

Participants were recruited by posting invitations to social media accounts, and from the Amazon MTurk and Prolific crowdsourcing apps (and restricted to U.S. citizens) during Spring and Summer 2023. The invitation directed participants to an anonymous 76-item online questionnaire that would take approximately 13 minutes and investigated relations between measures of SM use, personality and attachment traits, and social interaction. We discarded 22% (*n* = 156) of the original cases due to incomplete responding, or those that failed attention check items. The final data set included a total of 555 participants (23.1% from social media, 40.9% from MTurk, 36.0% from Prolific) for which there were no missing data on scale items. The ages of participants ranged from 18–79 (*M* = 39.8, *SD* = 13.5). The sex distribution was male (47.1%), female (51.8%), and non-binary (1.1%). The ethnic identity distribution was White (76.8%), African-American (9.9%), Latino-American (4.7%), Asian-American (5.9%), and other/mixed ethnicity (2.3%). The study was approved by the University Institutional Review Board (Reference #: 2023-04-01, April 5, 2023) and a statement of informed consent was included in the online questionnaire.

### Measures

**Attachment.** We measured attachment-related anxiety and avoidance using the ECR-12 [8]. Anxiety and avoidance are measured with six items each, rated on a Likert scale (1 = *strongly disagree* to 7 = *strongly agree*). The reliability of the scales in the present study was excellent (Cronbach’s α: anxiety = .90, avoidance = .90, McDonald’s ω: anxiety = .90, avoidance = .91). Higher scores indicate greater anxiety and avoidance.

**Grandiose narcissism.** We measured overt or GN using the narcissism scale of the Short Dark Triad (SD3) [24]. The SD3 measures narcissism with 9 items rated on a Likert scale (1 = *strongly disagree* to 5 = *strongly agree*). The reliability of the scale in the present study was good (Cronbach’s α = .81, McDonald’s ω = .82). Higher scores indicate greater grandiose narcissism.

**Vulnerable narcissism.** We measured covert or VN using the Hypersensitive Narcissism Scale (HNS) [16]. The HNS measures narcissism with 10 items rated on a Likert scale (1 = v*ery uncharacteristic or untrue, strongly disagree* to 5 = *very characteristic or true, strongly agree*). The reliability of the scale in the present study was acceptable (Cronbach’s α = .79, McDonald’s ω = .79). Higher scores indicate greater vulnerable narcissism.

**Interpersonal competence.** We measured IC using the Interpersonal Competence Questionnaire (ICQ-15) [93], which includes 15 items measured with 3 items each rated on a Likert scale (1 = *I’m poor at this; I’d feel so uncomfortable and unable to handle this situation, I’d avoid it if possible* to 4 = *I’m EXTREMELY good at this; I’d feel very comfortable and could handle this situation very well*). In the present analyses we use the full 15-item scale score computed as the mean of all 15 items. The reliability of the 15-item scale in the present study was good (Cronbach’s α = .88, McDonald’s ω = .88).

**Problematic social media use.** We measured PSMU using 10 original items rated on a Likert scale (1 = *strongly disagree* to 7 = *strongly agree*). Items were written largely based on the criteria used in the DSM-5 regarding alcohol use disorder [94]. The Kaiser-Meyer-Olkin (KMO) measure verified the sampling adequacy for the EFA (KMO = 0.914), Bartlett’s Test of Sphericity indicated the inter-item correlations were sufficiently large for factor analysis (χ^2^ (45, *N* = 277) = 1418, *p* < .001), and Average Variance Extracted (AVE = 0.54) supported the convergent validity of the items. We used a split-half cross-validation procedure to create the PSMU scale. After randomly splitting the full sample in half, an exploratory factor analysis (EFA) with ML estimation and varimax rotation resulted in two-factors with good overall model fit (χ^2^ (26, *N* = 277) = 66.1, *p* < .001, TLI = 0.95, RMSEA = 0.08 (90% CI [0.05, 0.10])). Given our focus on measuring PSMU as a one-dimensional construct, we then used the six items that loaded strongly only on factor 1 (all factor loadings > 0.50) and computed a Confirmatory Factor Analysis (CFA) with full information maximum likelihood (FIML) estimation on the other half of the sample. The CFA results indicated a mixed fit: acceptable according to CFI, TLI, and SRMR, but weaker fit according to RMSEA (χ^2^ (9, *N* = 278) = 32.5, *p* < .001, CFI = 0.97, TLI = 0.95, SRMR = 0.03, RMSEA = 0.10 (90% CI [0.06, 0.13])). Finally, we computed a CFA with FIML estimation on the full sample, using the same six items, which also resulted in an overall acceptable model fit (χ^2^ (9, *N* = 555) = 49.0, *p* < .001, CFI = 0.97, TLI = 0.96, SRMR = 0.03, RMSEA = 0.09 (90% CI [0.07, 0.12])). We then computed a PSMU scale score as a mean of these six items. The six retained items stated: “I sometimes find that I have spent more time on social media than I planned or intended.” “I have tried to limit or quit using social media, but that never lasts very long.” “I use social media when I know it is not a good idea to do so.” “I use social media when I’m feeling down or to avoid thinking about something that’s bothering me.” “I spend more time using social media than I used to.” and “I often check my social media impulsively, for no reason.” The four items that were not retained stated: “I tend to organize my time and activities around being able to use social media.” “I have trouble keeping my mind off using social media.” “I don’t do as well as I could at school or work because of my social media use.” and “I get anxious or disturbed when I can’t use social media.” The reliability of the 6-item scale in the present study was good (Cronbach’s α = .88, McDonald’s ω = .88). Higher scores indicate greater PSMU.

### Statistical analyses

Data analyses were performed using R (version 4.3.2) and Jamovi (version 2.7.23.0). Scale reliabilities were assessed using Cronbach’s alpha and McDonald’s omega (≥ 0.90 = excellent, 0.80 to 0.89 = good, 0.70 to 0.79 = acceptable). Adequacy of the data used to construct the PSMU scale was assessed using the Kaiser-Meyer-Olkin (KMO) measure (≥ 0.9 = excellent), Bartlett’s Test of Sphericity (*p* ≤ .05 recommended), and Average Variance Extracted (≥ 0.50 recommended). EFA and CFA models were assessed using well-established fit indices and cutoffs: CFI and TLI (≥ 0.95 = good), SRMR (≤ 0.05 = good), and RMSEA (≤ 0.08 = adequate/fair) criteria [95–97]. Bivariate correlations were used to test Hypotheses 1–7. Pearson’s correlations were used to assess associations between pairs of variables (small (.10 –.29), medium (.30 –.49), and large (≥.50) effect sizes). In order to test H8 we used LPA, computed using the TidyLPA package in R [98] with the Expectation-Maximization (EM) algorithm estimation protocol. Models were estimated with free parameters for both variances and covariances across all profiles (Model convergence issues were encountered when additional parameter constraints on the covariance structure were imposed for some of the LPA models.). LPA is a person-centered (rather than variable-centered) approach in which subgroups of participants are identified as belonging to separate groups or profiles of a categorical latent variable if they demonstrate within-group homogeneity and between-group heterogeneity on levels of the LPA indicator variables [92]. Our indicator variables are attachment anxiety, attachment avoidance, GN, VN, and IC. Our sample size exceeded the recommended minimum sample size of 500 for identifying the correct number of latent profiles [99]. The choice of the best profile solution is determined (a) by computing a number of models with increasing numbers of profiles and comparing them on various measures of model fit and, more importantly, (b) the theoretical, substantive interpretability of profiles [100]. Thus, if two different profiles are similarly distinctive based on quantitative results, choosing the best model relies on the theoretical interpretability of the profiles [92]. We report the commonly used LPA model fit indices: log-likelihood values (L-L), Bayesian Information Criterion (BIC) and Sample-size Adjusted BIC (SABIC), Akaike Information Criterion (AIC), Entropy and pMin (average minimum probability of assignment to group profiles), and Bootstrapped Likelihood Ratio Test (BLRT, and its *p* value). Better model fit is indicated by lower values for BIC, SABIC, and AIC values, and higher values for L-L, entropy and pMin (reflecting confidence in the assignment of participants to the correct profile) and BLRT. A common recommended guideline is to choose the model after which the BLRT is statistically non-significant for the first time [100]. Recommendations also include a minimum Entropy level of.60 [92,101] and a minimum pMin of.70 [102,103]. It is also recommended to provide additional criterion-related validity evidence for the LPA model [92] by testing mean differences between participants in different profiles on an auxiliary variable. The auxiliary variable in the present study is PSMU which was tested using ANOVA. We used z-standardized mean scale scores for the five indicator variables and the auxiliary variable in the LPA and ANOVA analyses to provide a clear interpretation and presentation of which values were above or below the sample mean [92]. Materials, data file, and reproducible code are available in the OSF repository https://osf.io/7ga2e/.

## Results

### Univariate and bivariate analyses

An exploration of the univariate distributions flagged fewer than 3% of the data as outliers on all LPA variables, which have fixed lower and upper limits and are, hence, legitimate data points. A careful examination of the outliers revealed a distinctive pattern between VN and IC that justified retention of these outliers in the data set. Thus, we retained all cases in the analyses.

Means and standard deviations for all scale variables are presented in Table 2. Results of bivariate correlation analyses testing H1-H7 (Table 2) indicated support for five of the first seven hypotheses and partially supported an additional hypothesis. Attachment anxiety and VN were positively correlated (*r*(553) =.57, *p* < .001) supporting H1. Attachment avoidance and GN were negatively correlated (*r*(553) = *–*.21, *p* < .001), failing to support H2. Attachment avoidance (*r*(553) = *–*.51, *p* < .001) and attachment anxiety (*r*(553) = *–*.22, *p* < .001) were negatively associated with IC, supporting H3. We found positive associations between PSMU and attachment anxiety (*r*(553) =.49, *p* < .001) and attachment avoidance (*r*(553) =.13, *p* = .002), with a stronger association between anxiety and PSMU, supporting H4. VN and IC (*r*(553) = *–*.47, *p* < .001) were negatively correlated, supporting H5. GN (*r*(553) =.08, *p* = .065) was not significantly correlated with PSMU, while VN (*r*(553) =.42, *p* < .001) was positively correlated with PSMU, partially supporting H6. IC was negatively correlated with PSMU (*r*(553) = *–*.18, *p* < .001), supporting H7.

**Table 2:** Mean scores and standard deviations, reliabilities, and correlations between the study variables.

Variable	Mean (*SD*)	α/ω	1	2	3	4	5
1. Anxiety	3.73 (1.63)	.90/.90					
2. Avoidance	2.99 (1.34)	.90/.91	.27***				
3. Grandiose Narcissism	2.58 (0.74)	.81/.82	.04	−.21***			
4. Vulnerable Narcissism	2.80 (0.70)	.79/.79	.57***	.32***	−.08		
5. IC	2.64 (0.50)	.88/.88	−.22***	−.51***	.50***	−.47***	
6. PSMU	3.71 (1.50)	.88/.88	.49***	.13**	.08	.42***	−.18***

*Note*. IC = Interpersonal Competence Questionnaire total scale score. PSMU = Problematic Social Media Use scale. α = Cronbach’s alpha. ω = McDonald’s omega. * *p* < .05, ** *p* < .01, *** *p* < .001

### Latent profile analysis

Results of the LPA are presented in Table 3. A variety of the fit statistics—coupled with a theoretically sound interpretation—supported a 4-profile solution. The 4-profile solution (a) was the model after which the BLRT became non-significant for the first time, (b) produced entropy (.646) and pMin (.707) values above the minimum recommended levels, and higher L-L and lower AIC values than the 3- or 2-profile models. In addition, in line with the theoretical perspective of the current study, the 4-profile solution produced profiles that clearly distinguish between secure, avoidant, anxious, and fearful attachment patterns based on the anxiety and avoidance dimensions of the ECR-12. Thus, the 4-profile solution provided a number of both quantitatively and qualitatively distinct profiles that satisfied the theoretical purpose of the present study and were substantially unique in their combination of different levels of the indicator variables, providing “new and theoretically interesting information” [92 p. 13] regarding the characteristics examined in the current study.

**Table 3:** Model fit information for latent profile analysis.

No. of Profiles	L-L	FP	AIC	BIC	SABIC	Entropy	pMin	BLRT	BLRT (*p*)
2	−3499	35	7080	7257	7127	0.749	0.874	138.0	0.0099
3	−3465	55	7054	7322	7125	0.625	0.793	68.1	0.0198
**4**	**−3435**	**75**	**7035**	**7349**	**7130**	**0.646**	**0.707**	**60.8**	**0.0495**
5	−3402	95	7012	7461	7131	0.690	0.749	65.4	0.0693

*Note:* Bold indicates the selected model. L-L = log likelihood; FP = free parameters; AIC = Akaike Information Criterion; BIC = Bayesian Information Criterion; SABIC = sample size adjusted BIC; Entropy = classification accuracy; pMin = lowest average probability of correct classification across all the profiles; BLRT = bootstrapped likelihood ratio test.

The LPA classified participants into four profiles (Fig 1), reflecting the secure, avoidant, anxious, and fearful attachment styles, as indicated by levels of the avoidant and anxious dimensions of the ECR-12. The secure profile comprised low scores on anxiety and avoidance, as well as a level of GN slightly above average, a very low level of VN, and a high level of IC. The avoidant profile comprised a high score on avoidance and a moderately low score on anxiety, as well as very low scores on GN and IC, and moderately high levels of VN. The anxious profile comprised a high score on anxiety and a moderately low score on avoidance, as well as moderately high scores on GN, VN, and IC. The fearful profile comprised very high scores on anxiety, avoidance, and VN, a moderately low score on GN, and a very low score on IC. H8 predicted that an LPA would result in profiles reflecting the four traditional attachment styles (which was supported), and levels of avoidance, anxiety, GN, VN, and IC within the profiles that concur with H1-H7. Anxiety and VN were positively aligned in three of the four profiles, supporting H1. Avoidance and GN were positively aligned in two of the four profiles, partially supporting H2. Avoidance was negatively aligned with IC in all four profiles, and anxiety was negatively aligned with IC in two of the four profiles, largely supporting H3. VN was negatively aligned with IC in three of the four profiles, largely supporting H5. Thus, within the profiles and indicator variables, we conclude that H8 is largely supported.

**Fig 1:**
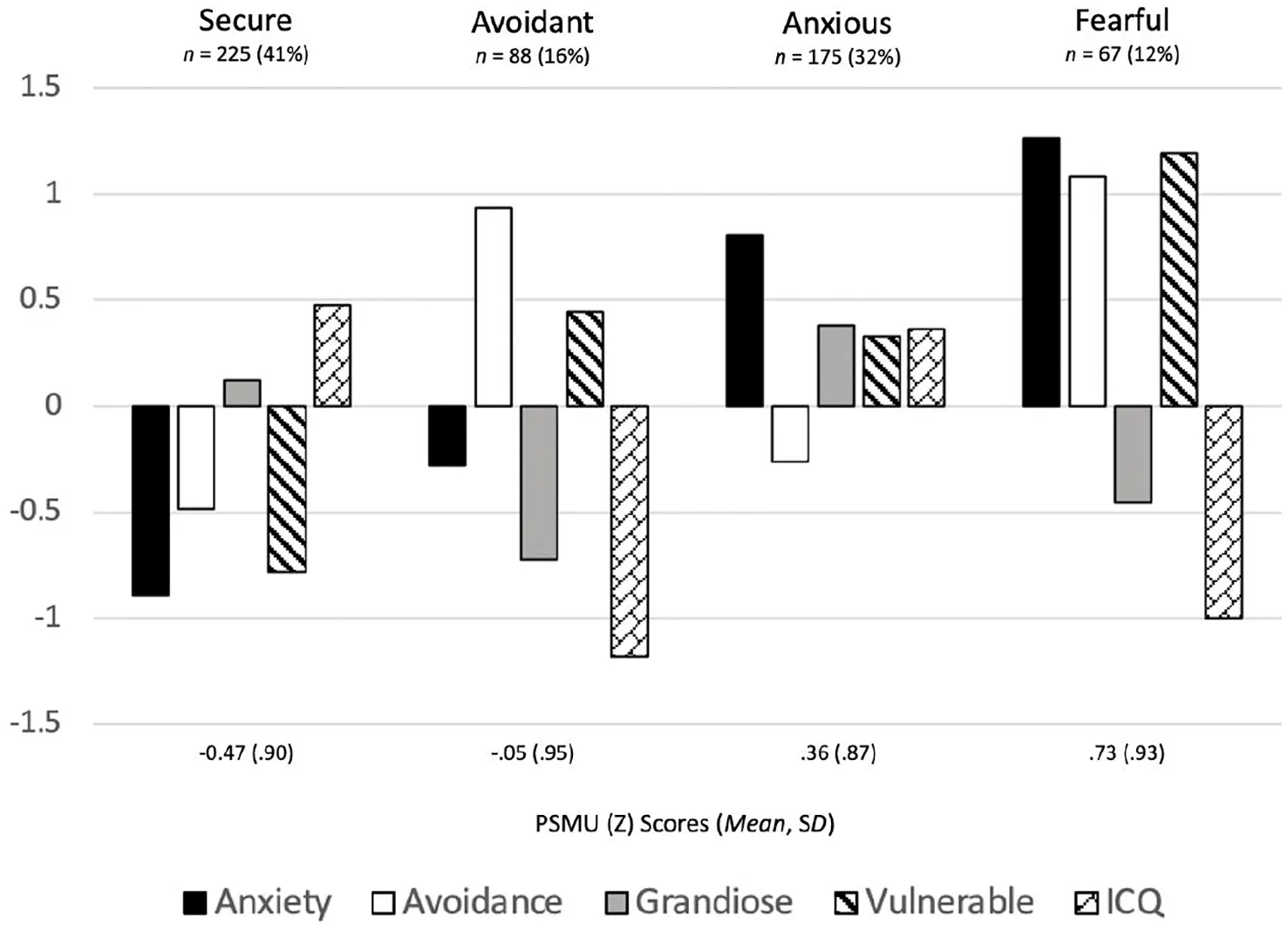
Standardized means for indicator variables across latent profiles with associated profile labels, profile sizes, and standardized mean scores on auxiliary variable. *Note:* Profiles are arranged from lowest (left) to highest (right) on profile mean scores for z-transformed PSMU (problematic social media use).

### Auxiliary analysis

We predicted (H9) that a secure profile would be associated with the lowest level of PSMU, and the anxious, avoidant, and fearful profiles would be associated with higher levels of PSMU. An ANOVA comparing PSMU between the four profiles was significant, *F*(3, 551) = 44.5, *p* < .001 (Table 4 and Fig 2). The secure profile demonstrated the lowest level of PSMU while, in ascending order, the avoidant, anxious, and fearful profiles demonstrated increasingly higher levels of PSMU. Tukey Honestly Significant Difference post-hoc comparisons of mean PSMU scores between each pair of profiles revealed that PSMU levels were significantly different between all of the profile comparisons (see Table 4). Thus, the ANOVA validation analysis supported H9. We expand upon the description of the profiles and associated levels of PSMU in the discussion below.

**Table 4:** Means, standard deviations, one-way ANOVA, and post-hoc comparisons of problematic social media use between LPA profiles.

Group	Mean	SD	*F*/*t*-test	*p*-value	Effect size	95% CI
Secure	−0.47	0.90	vs Avoidant *t* = −3.71	.001	*d* = −0.47	−0.72, −0.22
			vs Anxious *t* = −9.15	<.001	*d* = −0.92	−1.12, −0.72
			vs Fearful *t* = −9.57	<.001	*d* = −1.33	−1.61, −1.06
Avoidant	−0.05	0.95	vs Anxious *t* = −3.49	.003	*d* = −0.46	−0.71, −0.20
			vs Fearful *t* = −5.34	<.001	*d* = −0.87	−1.19, −0.54
Anxious	0.36	0.87	vs Fearful *t* = −2.85	.023	*d* = −0.41	−0.69, −0.13
Fearful	0.73	0.93				
Overall ANOVA			*F*(3, 551) = 44.5	<.001	η^2^ = 0.20	

*Note:* Post-hoc comparisons performed using Tukey HSD. Mean scores are standardized *z*-scores.

**Fig 2:**
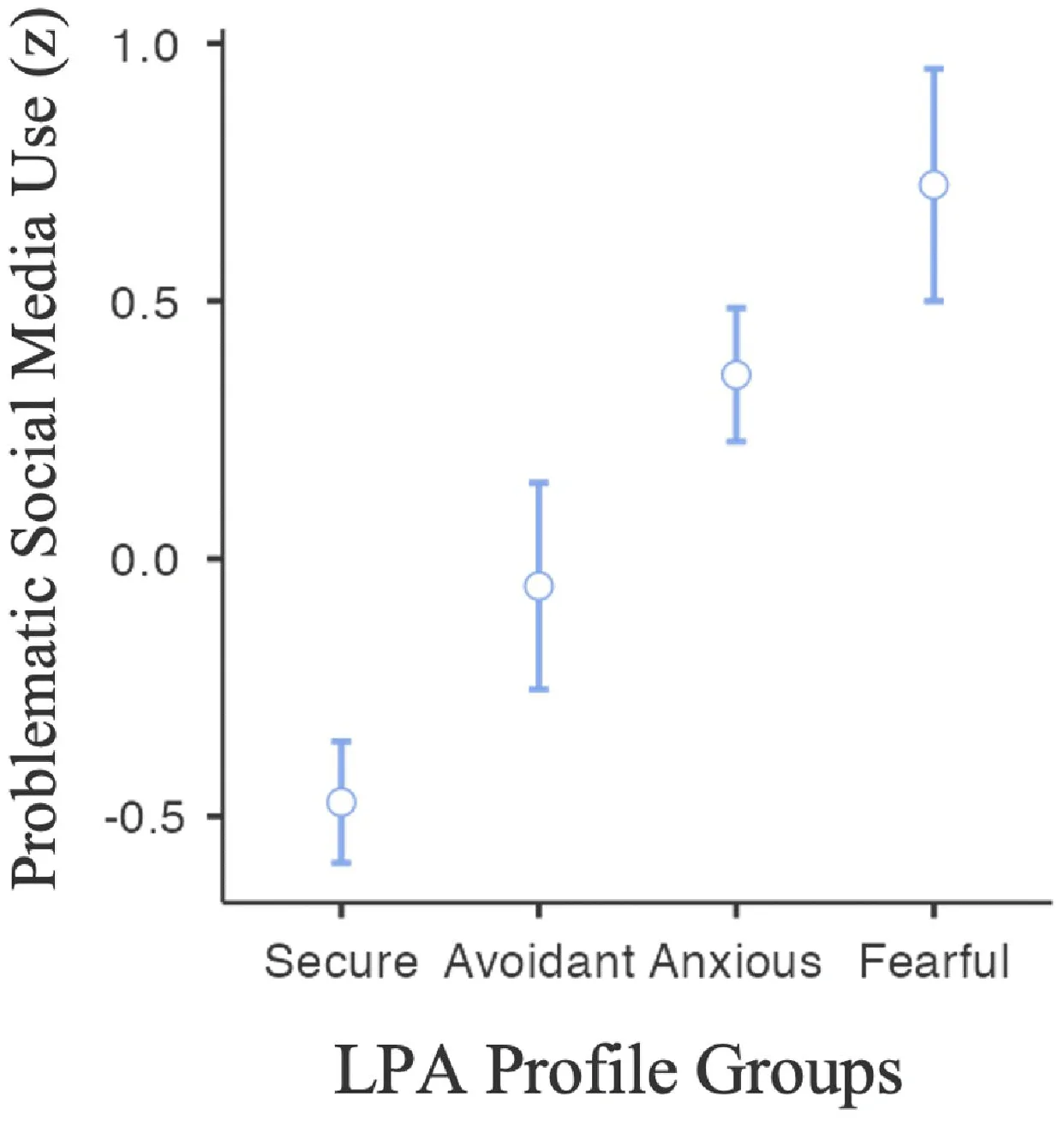
Plot of post-hoc comparisons of LPA profile groups on standardized problematic social media use. *Note:* Profiles are arranged from lowest (left) to highest (right) on profile mean scores for z-transformed PSMU (problematic social media use).

We did not predict, based on existing literature, sex or age differences among latent profiles. Nonetheless, as a further exploration of the patterns amongst the obtained profiles we examined sex and age differences using a chi-square test of independence and a one-way ANOVA, respectively. A chi-square test of independence examining the relationship between sex and profile membership indicated a significant association between the two variables (χ^2^ (3, 548) = 14.0, *p* = .003) with more males than females in the secure profile, and more females than males in the anxious profile. A one-way ANOVA revealed a significant age difference between profiles, *F*(3, 541) = 22.1, *p* < .001, η^2^ = 0.11. Post-hoc comparisons using the Tukey HSD method indicated that participants in the secure (*M* = 44.2, *SD* = 13.4) and avoidant (*M* = 42.2, *SD* = 12.5) profiles were significantly older than participants in the anxious (*M* = 34.8, *SD* = 12.5) and fearful (*M* = 34.5, *SD* = 12.0) profiles (both *p* ≤ .002).

## Discussion

Our study was primarily focused on the predictions that a person-centered analysis would reveal four distinct groups of participants demonstrating levels of attachment anxiety and attachment avoidance that correspond to the secure, avoidant, anxious, and fearful attachment styles, the profiles would be characterized by predicted levels of GN, VN, and IC, and the profiles would also be distinguished regarding participants’ levels of PSMU, with lower PSMU levels associated with a secure attachment style and higher PSMU levels associated with avoidant, anxious, and fearful attachment styles. The results support both predictions, suggesting that while persons with an anxious attachment style are prone to PSMU, persons with a fearful attachment style, with high levels of anxiety, avoidance, and VN, are most likely to express PSMU. Below we briefly interpret the variable-centered results, followed by an extensive interpretation of the person-centered results. Lastly, we touch on the exploratory analyses of profile differences by age and sex.

Full-sample correlational patterns indicate overall support for our hypotheses and similarities to the general pattern of results of previous studies. H1: A tendency toward VN was strongly associated with a high degree of anxious attachment, indicating a tendency for persons with a hypersensitive, low self-concept, introverted style of narcissism to worry more about their relationship with a partner [33]. H2: The predicted positive association between avoidance and GN was not supported. This may reflect the somewhat inconsistent results reported in previous studies regarding avoidance and GN [21,35]. While the obtained correlation was small, in the present sample avoidance was significantly negatively associated with GN. At least in our sample as a whole, an overt, attention-seeking style of narcissism was to some degree more prominent among persons who did not report avoiding closeness in their relationships with romantic partners. H3: Both high avoidance and high anxiety predicted low IC and, consistent with previous research [43,44], IC was more strongly associated with avoidance than anxiety. Those with high anxiety, perhaps lacking sufficient trust in relationships, may simply judge their interpersonal skills negatively. Those with high avoidance may be conditioned internally to eschew opportunities for interpersonal connectedness [104] and thereby fail to develop a positive interpersonal self-concept. H4: PSMU was associated weakly with avoidance but more strongly with anxiety. This is consistent with existing literature and suggests PSMU may reflect a tendency among anxiously attached individuals to compensate for unsatisfactory face-to-face interpersonal needs through on-line interaction [55]. H5: Higher VN was associated with lower IC, suggesting that the internal emotional instability and distress characteristic of VN is manifested in interpersonal impairment [39,71]. H6: GN was not significantly associated with PSMU, indicating that persons with high levels of GN do not feel compelled to use SM in a manner reflected on PSMU measures to engender admiration from others. In contrast, VN was strongly positively associated with PSMU, suggesting that while those with a high level of VN may use SM to solicit relationship attention that they find difficult to achieve in real life, given their tendency toward hypersensitivity and shyness [80,83]. H7: Lastly, IC and PSMU were negatively correlated, supporting previous evidence that problematic use of smartphones may detrimentally affect social skills [89, 90].

The results of the LPA suggest that, as predicted (H8), attachment anxiety, attachment avoidance, GN, VN, and IC together provide a distinction between four different profiles or groups within the sample, each characterized by a unique combination of levels of the five indicator variables. Following our premise that attachment style may underlie the development of narcissistic tendencies and IC, we predicted that a person-centered analysis would reveal separate profiles reflecting each of the four traditional attachment styles (secure, anxious, avoidant, fearful), which we can infer from levels of anxiety and avoidance. The LPA results support this prediction. While the pattern of positive and negative bivariate associations was in some ways mirrored in the combination of variable levels in the obtained profiles, the variable levels were different across profiles and there were some variable combinations that did not mirror the bivariate results. In fact we would not expect variable combinations in all profiles to follow the exact pattern found in the bivariate correlations. This is the general purpose and benefit of the person-centered analytic approach provided by LPA, to investigate the manner in which characteristics are reflected in different ways in different groups within a larger sample. Thus, we turn to our interpretation of the LPA results followed by the validation of the LPA by comparing mean profile scores on our auxiliary variable, PSMU. For ease of interpretation of the profiles and their relation to PSMU, the profiles in Fig 1 are ordered from lowest (left) to highest (right) PSMU mean score.

As shown in Fig 1, the first profile, labeled Secure, was characterized by levels of the indicator variables we would expect from securely attached individuals: low levels of anxiety, avoidance, and VN, and a high level of IC. IC was in fact higher in this group than the other three profiles, as expected. We suggest that the high level of IC, low anxiety, low avoidance, and low VN, and slightly above-average GN reflect characteristics we would expect from securely attached individuals characterized by healthy psychological and interpersonal well-being [8,104].

The second profile, labeled Avoidant, was characterized by levels of the indicator variables we would expect from individuals with an avoidant or dismissing attachment style: high avoidance and VN, and low anxiety, GN, and IC. These avoidant individuals were characterized by the reactive (VN) rather than bold (GN) type of narcissism [15]. Dominated by a high level of avoidance, this group also demonstrated the lowest level of IC, congruent with previous studies [44,47] and echoing prior research indicating avoidant individuals tend to hold high expectations for failure in relationships [105].

The third profile, labeled Anxious, was characterized by a pattern of indicator variable levels that noticeably deviates from results found in earlier studies. This group was characterized by high anxiety, moderately high GN, VN, and IC, and moderately low avoidance. Previous research generally reports negative associations between anxiety and IC [44,45] and negative relations between VN and IC [32,106]. The results of our LPA identified two types of ‘high anxiety’ individuals; those in the anxious profile who reported not only high anxiety and moderately high VN, but also moderately high GN. For participants in the anxious profile, high anxiety (but low avoidance) may contribute to moderately high levels of narcissistic tendencies, and low avoidance and moderately high GN may contribute to a moderately high interpersonal self-concept, all serving to solicit more attention from others. Indeed IC was higher in this profile than any other except for the secure profile. This pattern contrasted with the second ‘high anxiety’ pattern observed in the fourth profile, which we labeled Fearful.

The Fearful profile was comprised of participants reporting high levels of anxiety, avoidance, and VN. The combination of high anxiety and high avoidance reflects the classic fearful or disorganized attachment style [5]. They also reported a moderately low level of GN and a very low level of IC. Our results support previous research indicating a fearful attachment style is associated with interpersonal difficulty in adulthood [38], especially in those with high VN [39]. Whereas the anxious profile was characterized by moderately high IC, the fearful profile was characterized by very low IC. In addition, the anxious and fearful profiles also differ on levels of avoidance, GN, and VN. Our results suggest, therefore, that the commonly reported negative association between anxiety and IC may apply specifically to participants who also experience high levels of avoidance and VN, and low GN, which in our study characterized persons in the fearful profile but not the anxious profile. Thus, high anxiety per se may not predict low IC, as commonly reported. Of the studies we found examining associations between attachment style and IC, the majority did not examine the fearful attachment style, even though most used the two-dimensional ECR [41–46,48,49]. Only three studies reported associations between all four attachment styles and some type of IC [40,50,51]. In our study, participants with high anxiety and moderately high GN and VN but low levels of avoidance reported moderately high IC, while participants with very high levels of anxiety, avoidance, and VN reported very low IC. The person-centered LPA method, indicating separate profiles for anxious and fearful participants, thus provides an important discrimination between characteristics associated with these two attachment styles.

Our auxiliary analysis (H9) compared the mean scores on PSMU across the four profiles revealing that, as predicted, securely attached participants demonstrated lower PSMU levels than avoidant, anxious, and fearful participants. A secure attachment style, characterized by a high level of trust in close relationships, may protect individuals against the need to rely on SM to satisfy relationship needs [53,55]. An avoidant style, however, coinciding in our sample with a moderately high level of VN, is associated with a higher level of PSMU than secure participants, suggesting that individuals who tend to expect relationship rejection, yet still desire attention and social affirmation, may gravitate more toward PSMU to temper and perhaps override the discomfort of real-life relationship dissatisfaction. Given the ease with which one can dismiss or detach from online interactions and relationships, avoidant individuals may perceive SM interactions less threatening and more controllable [107]. Interestingly, our results may suggest an explanation for previous reviews and meta-analytic results indicating an inconsistent association between avoidance and PSMU [52,55], as the avoidant profile was significantly higher on PSMU than the secure profile yet significantly lower on PSMU than the fearful profile, (which also demonstrated a high level of avoidance). Many if not most prior studies often have not measured (or classified participants in) the fearful attachment style [37,108]. In some studies, therefore, some participants may have been misclassified as avoidant rather than fearful. Thus, the inconsistency in previous studies may also suggest a limitation of variable-centered analyses.

As indicated elsewhere [53,58], we found that PSMU was more strongly related to anxiety than avoidance, as PSMU levels were significantly higher among the anxious and fearful profiles. High attachment anxiety, reflecting fear of rejection and perceived unreliability of support from others, may prompt individuals with a fragile or timid self-concept and/or unmet attachment needs [55] to engage in excessive or hyperactive [55] reassurance-seeking in a context that is both more readily available and in some ways less threatening: smartphone and SM use [59,109,110]. In addition, the highest PSMU level was evident among fearful participants, in contrast to some previous research. However, one of the previous studies reported low reliability coefficients of the attachment measure employed [64], and another study used an older instrument that measured levels of the four attachment styles using self-reported endorsement of a single item for each attachment style [65]. Our results, showing that a fearful attachment style was associated with the highest level of PSMU, concur with a previous study that also measured attachment using the ECR [59]. The stronger association between PSMU and the ECR (compared to other attachment measures) has been noted elsewhere [55]. The fearful attachment style, characterized by both a fear of reliance on others and a strong desire for reassurance, may prompt individuals to seek comfort for relationship distress on SM yet fail to perceive that contact through SM is sufficiently reassuring or comforting. This tendency toward high PSMU may be enhanced among those with a high level of VN, given their levels of shyness, shame regarding interpersonal needs, and hypersensitivity [39].

From a practical standpoint, these findings suggest that avoidant attachment alone, while associated with very low IC, is not associated with high PSMU. Our results indicate that attachment anxiety is the key component associated with PSMU, and persons with high-attention-seeking tendencies are more likely to express PSMU. Of practical use to researchers, clinicians, prevention programs (etc.) these results suggest individuals who demonstrate both high attachment anxiety and attachment avoidance (shy, ashamed of relationship needs, hypersensitive to criticism) may be at greater risk of failing to express necessary interpersonal skills and may instead attempt to satisfy relationship needs via PSMU. Finally, although not a focus of our research, we explored potential sex and age differences among profiles, finding that there were more males than females in the secure profile and more females than males in the anxious profile. A meta-analysis of studies prior to 2011 indicated that males tended to be higher on avoidance and females higher on anxiety [111]; our results are similar regarding anxiety but not avoidance. Our results also indicated that persons in the secure and avoidant profiles were older than persons in the anxious and fearful profiles. By comparison, one study of over 86,000 internet respondents found that anxiety is higher among younger adults, and avoidance is highest in middle adulthood [112]. Thus, our results again suggest similarity with prior research regarding anxiety but not avoidance. However, one of the reasons we did not expect or hypothesize age and sex differences that correspond to prior studies is that we were primarily interested in the pattern of attachment style profiles that also included GN, VN, and IC. We suggest that our results may help to inform future studies designed to more formally and directly test hypotheses regarding age and sex differences.

### Limitations and future research

First, while the premise of our study suggested that LPA profiles would be largely structured around the attachment anxiety and avoidance dimensions (as attachment styles may lead to the development of narcissism, both of which would then contribute to IC, all of which would then contribute to potential PSMU), we of course have no evidence of the causal relations or developmental sequence between these constructs. Such evidence could be illuminated by future studies that employ a longitudinal design. Second, while we expanded upon previous research by specifically examining both GN and VN in relation to attachment styles, IC, and PSMU, we did not measure the more recently identified communal narcissism (CN) [113] a sub-type of grandiose narcissism characterized by a desire to be seen by others in the community as being highly altruistic or ‘saintly.’ The limited existing evidence has demonstrated inconsistent associations between GN, VN, and CN and SM use and PSMU. One study [114] that compared GN, VN, and CN found that VN was a stronger predictor of SM use than either GN or CN, while another study [79] found that CN was a stronger predictor of SM addiction than GN (admiration or rivalry types) or VN. More research is needed to investigate these potentially conflicting results. Since our sample was limited to U.S. participants, it is possible that our results may not generalize to populations and cultures that are less focused on individuality rather than collectivism, or that CN may be more strongly associated with attachment styles, IC, and PSMU than GN or VN in collectivistic cultures. Third, our validation of the PSMU measure would have been strengthened by including, for comparison, a previously validated scale such as the Bergen Social Media Addiction Scale [115]. Finally, we have no way of knowing whether the profiles obtained in our sample would apply to other samples, either within the U.S. or outside of the U.S. LPA has yet to become a common data analytic method. Future studies employing LPA methods on the same variables with different samples would strengthen our understanding of the patterns among the variables in our study.

## Conclusions

A growing body of research examines the relations between attachment styles, narcissism, interpersonal skills, and PSMU. Yet, existing studies are somewhat limited by (a) a predominant focus on GN, (b) a failure to address the fearful attachment style, and (c) employing measures of SM use frequency or intensity rather than PSMU. To address these limitations, we examined associations between measures of attachment anxiety and avoidance, GN, VN, IC, and PSMU. Bivariate associations between variables generally concurred with the results of previous studies, with the exception of the negative association in our sample between avoidance and GN, and no significant association between GN and PSMU. The LPA produced separate profiles characterized by levels of anxiety and avoidance that distinguish the secure, avoidant, anxious, and fearful attachment styles. The four profiles were also distinguished by unique combinations of GN, VN, and IC, and suggest differences between the anxious and fearful attachment styles that have been overlooked in previous studies. We validated the LPA results by comparing profile mean scores on PSMU, revealing that secure participants reported the lowest PSMU, followed in increasing order of PSMU levels by avoidant, anxious, and fearful participants. Thus, PSMU appears most strongly associated with the fearful attachment style. The person-centered LPA approach thus offers a perspective that expands upon, and helps explain inconsistencies in, previous investigations of these constructs.
