# Supplementary material for: Attachment, grandiose and vulnerable narcissism, interpersonal competence, and problematic social media use: A latent profile analysis
Source: PLoS One. 2026 Oct 21;21(10):e0359569. doi: 10.1371/journal.pone.0359569 (PMC13686506; doi:10.1371/journal.pone.0359569)
Supplement: Supplementary file 1 [file pone.0359569.s002.docx]

We thank the reviewers for their thorough examination of our original manuscript. Their suggestions have indeed led us to revise the manuscript in ways that strengthen the results and text, and we hope will benefit readers.

Below are comments and suggestions from Reviewers 1 - 3, followed by our indented replies to each suggestion.

======================================

Reviewer #1: I would like to begin by highlighting several strengths of the manuscript in relation to the journal’s publication criteria. The study presents original empirical research that integrates theoretically relevant constructs often examined separately (attachment, grandiose and vulnerable narcissism, interpersonal competence, and problematic social media use). The analytical strategy is clearly described and combines variable-centered and person-centered approaches through correlational analyses and latent profile analysis. The sample size is adequate for the statistical procedures conducted, and model selection criteria and fit indices are reported transparently, allowing the reader to evaluate the analytical decisions. In addition, the availability of data, materials, and code in an open repository substantially enhances transparency and reproducibility, aligning with current standards for research integrity and data availability.

However, I would like to highlight several general issues that should be addressed before the manuscript can be considered suitable for publication.

First, although the hypotheses (H1–H9) are clearly stated, the overall analytical architecture lacks coherence. The manuscript combines variable-centered hypotheses, person-centered predictions through LPA, and a subsequent validation step examining differences in PSMU across profiles. While using both approaches is acceptable, the manuscript does not clarify how they relate to one another or which constitutes the primary objective of the study. It remains uncertain whether the central contribution lies in confirming established associations, identifying latent subgroups, or explaining variation in problematic social media use. As presented, the transition from correlational analyses to profile modeling and validation reads as sequential rather than theoretically driven. Clarifying the hierarchy of objectives and organizing the hypotheses accordingly would strengthen the internal logic of the study.

We have added a new opening paragraph in the Introduction that is designed to clarify the primary objectives of the study and explains that the variable-centered correlational analyses are of secondary importance and are primarily meant to provide support and context for the person-centered latent profile analysis and subsequent examination of profile differences in PSMU.

Second, the measurement of PSMU is a vulnerable aspect of the manuscript. The instrument was developed ad hoc based on DSM-5 criteria for alcohol use disorder and is not a previously validated measure of problematic social media use. After conducting an exploratory factor analysis, four items were removed and only six were retained, all within the same sample used for the main analyses. No cross-validation or confirmatory factor analysis was conducted to verify the retained structure, and no evidence of convergent, discriminant, or criterion validity is provided. The manuscript also does not compare this measure with established instruments such as the Bergen Social Media Addiction Scale or the Social Media Disorder Scale, both validated in U.S. populations. This limits the interpretability of the findings and makes it difficult to situate the results within the existing literature. Because PSMU is used to validate the latent profile solution, greater attention to its measurement is necessary. This limitation should be clearly acknowledged.

We addressed the above concern in our revision using cross-validation, explained in detail in the revised manuscript. We randomly split the sample and computed an exploratory factor analysis (EFA) on the first half. The EFA produced a factor containing the same six-items used in the original manuscript to create the PSMU scale. We then computed a confirmatory factor analysis (CFA) on the second half of the split file using the six items identified by the EFA. This CFA confirmed the factor structure of the EFA. Finally, we computed a CFA on the full sample using the identified six items, confirming the factor structure of the PSMU scale for the full sample. The EFA and CFAs demonstrated overall good model fits.

Regarding the use of an original set of items, rather than the Bergen Social Media Addiction Scale (BSMS), we submit that the original items and those retained in our PSMU scale are qualitatively quite similar to the BSMS items. More specifically, our PSMU items address characteristics of addiction (in this case, social media addiction, more commonly labeled problematic social media use in recent studies) that are also addressed by the items in the BSMS, namely relapse/loss of control, conflict/functional impairment, mood modification, and craving/tolerance.

Also, similar to Andreassen et al's. (2016) six-item scale, which was significantly correlated with anxiety (*r* = .34, *p* < .01), evidence of convergent validity of our six-item PSMU scale is reflected in the significant positive correlations between the PSMU and attachment anxiety (*r* = .49, *p* < .001) and vulnerable narcissism (*r* = .42, *p* < .001). Evidence of discriminant validity is reflected in the significant negative correlation between PSMU and interpersonal competence (*r* = -.18, *p* < .001).

Third, concerns also arise regarding item removal across several measures. One item was removed from the SD3, two from the HNS, and four from the PSMU scale, all within the same dataset subsequently used for the primary analyses. Removing items without cross-validation increases the likelihood that model fit improvements reflect sample-specific characteristics rather than stable structural properties. For validated instruments such as the SD3 and HNS, modifying the original structure requires stronger theoretical justification and discussion of the implications for construct coverage and comparability with prior research. As noted above, the absence of independent validation limits confidence in the stability of the revised scales. Clearer justification and acknowledgment of this limitation would strengthen the manuscript.

We originally considered our confirmatory approach (CFA) to demonstrate the psychometric properties in our study of previously published scales (for attachment anxiety and avoidance, grandiose and vulnerable narcissism, and interpersonal competence) to be a more stringent approach than relying solely on common measures of reliability, such as Cronbach's alpha. However, the critiques of reviewers regarding dropping scale items are reasonable. Thus, we reverted to the more common approach of computing and reporting Cronbach and McDonald's reliability coefficients for established scales. (We explained how we addressed the reviewer's concern regarding the PSMU scale above.)

After computing full-scale scores for grandiose narcissism and vulnerable narcissism, using all items in those published measures, we computed reliability coefficients for all scales and include them in the Measures section of our revised manuscript. We also re-ran all analyses, which resulted in an LPA model that indeed more strongly supports our original hypothesis (H8), predicting profiles reflecting the four traditional attachment styles. The revised LPA supports a four-profile structure, and the profiles reflect levels of attachment anxiety and attachment avoidance that characterize the four traditional attachment styles (secure, avoidant, anxious, and fearful). The profiles are also characterized by levels of grandiose narcissism, vulnerable narcissism, and interpersonal competence that largely support our predictions regarding relations between attachment styles and narcissism and interpersonal competence. Lastly, the validation of the profiles using ANOVA and post-hoc analyses to compare levels of (z-transformed) PSMU among the four LPA profiles demonstrated statistically significant differences between all four profiles, also supporting our original hypothesis (H9). The levels of PSMU across the four profiles also support and strengthen findings of previous studies indicating that PSMU is lower among securely attached adults, higher for avoidantly attached adults, and higher still for anxiously attached adults. Our results also extend previous literature by demonstrating PSMU is highest for adults with high levels of both anxious and avoidant attachment, which is the pattern that characterizes the fearful attachment style.

Specific comments

The abstract should state more clearly the primary objective of the study and the main conclusions derived from it.

We have revised the abstract as suggested.

The introduction is overly extensive and at times more descriptive than integrative. Although the literature review is thorough, the detailed presentation of numerous associations makes it difficult to identify the central focus of the study. A more selective synthesis emphasizing the most consistent findings would improve clarity. The introduction would also benefit from a concluding paragraph that explicitly states the primary objective before presenting the hypotheses.

We appreciate the reviewer's concern regarding the introduction. We have added a paragraph at the beginning of the introduction (at the suggestion of the present reviewer, above), as well as a concluding paragraph at the end of the introduction, that we believe integrate the separate sections of the introduction. Given the inherent complexity of the LPA, we believe that a concise but pointed examination of the associations between the different variables (three of which are only one paragraph in length) is useful. We spent considerable time identifying existing studies on each of the associations between the variables specifically to focus each section on the best available evidence. We hope that given the other structural / explanatory changes we have made to the manuscript, prompted by the expert suggestions of the reviewers, the introduction no longer seems as descriptive as in the original submission, and instead communicates a useful integration of the existing literature.

Given the number of constructs involved (attachment, grandiose narcissism, vulnerable narcissism, interpersonal competence, and PSMU), including a concise table summarizing their conceptual definitions and operationalizations would improve readability.

We have added a new Table 1 to the end of the Introduction summarizing the variables/constructs.

Consistency in abbreviations should be reviewed. CFA is introduced on page 10, yet the full term is later repeated alongside the abbreviation. Some scales are properly introduced with their full name followed by the abbreviation, while others, such as ICQ-15, appear without prior clarification. Standardizing abbreviations would improve clarity.

We have made the suggested corrections throughout the manuscript.

The use of italics should also be revised. In some instances, italics appear to be used for emphasis without a clear rationale, such as on page 23, line 489, where the words specifically and also are italicized. Formatting should be consistent and limited to standard academic conventions.

We have removed italics except for scale anchors.

The format of Table 1 should be aligned with the journal’s presentation guidelines.

We have revised the format of our Tables accordingly.

Finally, the discussion would benefit from a clearer structure. It would be helpful to restate the primary objective before interpreting the findings and to organize the discussion around the stated hypotheses, indicating which were supported. A more structured discussion would improve coherence between the introduction, hypotheses, and results.

We have revised the discussion as suggested above.

Reviewer #2: The study is well-written and interesting. However, I have some comments for authors to reconsider before publication.

HYPOTHESES

p.9. Hypotheses 8 should be shortened

We shortened H8 as suggested.

The sentence “Our results generally supported the hypotheses. “ could be omitted since the hypotheses will be tested in the results section and will be discussed in the next section-

We omitted the sentence noted above.

Discussion

In the Discussion sections authors should do a lot of work. An introductory paragraph should present the aim of the study and a summary of the findings. Then, start the discussion with the most important findings which depict the contribution of the study to the relevant literature.

We have revised the Discussion as suggested above.

On page 20 the information in the brackets should appear in the main text. The same throughout the manuscript.

We have corrected the manuscript as noted above.

My suggestion is the Discussion to start with the profiles with regard to the use of SM and incorporate where appropriate the information before the Latent Analysis, in the current version.
Practical implications should be of interest.

We have edited the manuscript as noted above.

Reviewer #3: Dear Authors, thank you for submitting the manuscript “Attachment, Grandiose and Vulnerable Narcissism, Interpersonal Competence, and Problematic Social Media Use: A Latent Profile Analysis”. The article falls into the scope of the journal, as it examines the associations between the two dimensions of attachment (anxiety and avoidance), grandiose and vulnerable narcissism, interpersonal competence, and problematic social media use among. Please find below some suggestions listed from the most important by sections:

Abstract

Please start the abstract with one sentence stating the present state of the subject presented and why it is important to investigate it further (in line 31). Also you could state firmly that it concerns a cross-sectional study, based on a self-reported survey.

We edited the abstract as suggested above.

Introduction

1) First, at the beginning of the literature review, it feels that the text opens rather immediately to the dimensions and research underscoring attachment, while some information about the concept itself is missing from the classical studies (i.e. a widely accepted definition/description of attachment). Furthermore, it would also be helpful to define shortly attachment dimensions, as well.

We added information as suggested above at the beginning of the Introduction.

2) The hypotheses section is detailed but could be reorganized. H1–H7 are correlational, whereas H8–H9 are person-centered and auxiliary-outcome hypotheses. Separating these into “variable-centered hypotheses” and “person-centered hypotheses” would improve clarity.

We edited the text at the beginning of the Hypotheses section to identify variable-centered vs person-centered hypotheses.

3) Line 200: Avoid discussing the results in the Introduction before presenting all evidence of your methodology, data and results.

We omitted the sentence as noted above.

Method

1) The most important concern concerns the PSMU measure. The authors used 10 original items based largely on DSM-5 alcohol use disorder criteria, conducted an EFA, retained six items from one factor, and then used this six-item score as the outcome/auxiliary variable. This decision needs more careful justification. The construct of problematic social media use is well established, and several validated measures are available. The use of a newly created, shortened, post hoc selected measure raises concerns about construct validity.

We addressed the above concern in our revision using cross-validation, explained in detail in the revised manuscript. We randomly split the sample and computed an exploratory factor analysis (EFA) on the first half. The EFA produced a factor containing the same six-items used in the original manuscript to create the PSMU scale. We then computed a confirmatory factor analysis (CFA) on the second half of the split file using the six items identified by the EFA. This CFA confirmed the factor structure of the EFA. Finally, we computed a CFA on the full sample using the identified six items, confirming the factor structure of the PSMU scale for the full sample. The EFA and CFAs demonstrated overall good model fits.

Regarding the use of an original set of items, rather than the Bergen Social Media Addiction Scale (BSMS), we submit that the original items and those retained in our PSMU scale are qualitatively quite similar to the BSMS items. More specifically, our PSMU items address characteristics of addiction (in this case, social media addiction, more commonly labeled problematic social media use in recent studies) that are also addressed by the items in the BSMS, namely relapse/loss of control, conflict/functional impairment, mood modification, and craving/tolerance.

Also, similar to Andreassen et al's. (2016) six-item scale, which was significantly correlated with anxiety (*r* = .34, *p* < .01), evidence of convergent validity of our six-item PSMU scale is reflected in the significant positive correlations between the PSMU and attachment anxiety (*r* = .49, *p* < .001) and vulnerable narcissism (*r* = .42, *p* < .001). Evidence of discriminant validity is reflected in the significant negative correlation between PSMU and interpersonal competence (*r* = -.18, *p* < .001).

2) Concerning the instruments, further evidence of CFA are required. Maybe also consider reporting information about data suitability for CFA, together with (AVE) and inter-factor correlations to evaluate convergent validity and the distinctiveness of the factors.

We added the following information to the Statistical analyses section: "Adequacy of the PSMU scale was assessed using the Kaiser-Meyer-Olkin (KMO) measure (≥ 0.90 = excellent), Bartlett's Test of Sphericity (*p* ≤ .05 recommended), and Average Variance Extracted (≥ 0.50 recommended)."

Also, we added the following information to the Measures (Problematic social media use) section: "The Kaiser-Meyer-Olkin (KMO) measure verified the sampling adequacy for the EFA (KMO = 0.914), Bartlett's Test of Sphericity indicated the inter-item correlations were sufficiently large for factor analysis (χ^2^ (45, *N* = 277) = 1418, *p* < .001), and Average Variance Extracted (AVE, 0.54) supported the convergent validity of the items."

As constructs were analyzed as aggregate measurements (as opposed to a multi-factor measurement model), inter-factor correlations are not an issue. Furthermore, our main scale (PSMU) focused on a single factor, which we believe is supported here (and strengthened by the critique of the reviewers).

Finally, you could report both McDonald’s omega and Cronbach’s alpha for internal consistency.

We added McDonald's ω to the manuscript, in the text and Table.

3) The grandiose narcissism scale was taken from the SD3, and one item was removed after CFA. The vulnerable narcissism scale was based on the HNS, and two items were removed after CFA. These item removals should be better justified. You should explain whether the decisions were theory-driven or purely statistical, whether modification indices guided them, and whether the resulting scales remain comparable to prior research.

This issue was also raised above by Reviewer #1, and we are grateful for this critique by two reviewers, as we believe the modified results have substantially enhanced the results of our study. Our response to this issue and summary of the modifications to the manuscript are restated here:

We originally considered our confirmatory approach (CFA) to demonstrate the psychometric properties in our study of previously published scales (for attachment anxiety and avoidance, grandiose and vulnerable narcissism, and interpersonal competence) to be a more stringent approach than relying solely common measures of reliability, such as Cronbach's alpha. However, the critiques of reviewers regarding dropping scale items are understandable. Thus, we reverted to the more common approach of computing and reporting Cronbach and McDonald's reliability coefficients for established scales. (We explained how we addressed the reviewer's concern regarding the PSMU scale above.)

After computing full-scale scores for grandiose narcissism and vulnerable narcissism, using all items in those scales, we computed reliability coefficients for all scales and include them in the Measures section of our revised manuscript. We also re-ran all analyses, which resulted in an LPA model that indeed more strongly supports our original hypothesis (H8), predicting profiles reflecting the four traditional attachment styles. The revised LPA supports a four-profile structure, and the profiles reflect levels of attachment anxiety and attachment avoidance that characterize the four traditional attachment styles (secure, avoidant, anxious, and fearful). The profiles are also characterized by levels of grandiose narcissism, vulnerable narcissism, and interpersonal competence that largely support our predictions regarding relations between attachment styles and narcissism and interpersonal competence. Lastly, the validation of the profiles using ANOVA and post-hoc analyses to compare levels of (z-transformed) PSMU among the four LPA profiles demonstrated statistically significant differences between all four profiles, also supporting our original hypothesis (H9). The levels of PSMU across the four profiles also support and strengthen findings of previous studies indicating that PSMU is lower among securely attached adults, higher for avoidantly attached adults, and higher still for anxiously attached adults. Our results also extend previous literature by demonstrating PSMU is highest for adults with high levels of both anxious and avoidant attachment, which is the pattern that characterizes the fearful attachment style.

4) You also need to use the same level of interpretation of reliability indices (e.g. line 226, it is stated as “good” (a = .90), but in line 252 a=.88 is interpreted as “excellent”. Consider reporting the cut-off scores in the Data Analysis section.

We made the above corrections and additions to the manuscript.

5) It would be helpful to report the versions of the statistical platforms that you used (i.e. R and Jamovi)

We added this information to the manuscript.

6) In the Statistical Analysis you go to lengths to describe the LPA (which is very helpful and commendable), but information about EFA, CFA and correlations are missing (e.g. cut-offs, model fit assumptions). Also, how you treated possible outliers.

We added information regarding EFA and CFA model fit assumptions/cutoffs, and correlation effect sizes, to the manuscript.

An exploration of the univariate distributions flagged fewer than 3% of the data as univariate outliers on all LPA variables, which have fixed lower and upper limits and are, hence, legitimate data points. A careful examination of the outliers revealed a distinctive pattern between the vulnerable narcissism and interpersonal competence scales that justified retention of these outliers in the data set as key person-variable attributes of the data set.. Thus, we retained all cases in the analyses.

We thank the reviewers for the helpful feedback, which steered us toward a model that more clearly captured the distinctive patterns/profiles in the data set.

Results

1) The text about the correlation results repeats Table data, which makes it redundant. You could highlight the most interesting findings, instead, referring to the Table for indices inspection.

We have been careful to limit our Results text to the data that support or fail to support H1 - H7, recognizing that *r* and *p* values are also normally reported in the text.

2) In lines 320-321 it is mentioned: “The LPA was computed using the TidyLPA package in R [95] with the EM algorithm estimation protocol.” This could be more fittingly be moved into the Statistical Analyses section and leave only the results in the present section.

We moved the suggested information above and the following sentence to the Statistical Analyses section. "Models were estimated with free parameters for both variances and covariances across all profiles. "

3) Furthermore, it should also be clarified how the exact TidyLPA model is specified. The statement that models “accounted for variable variances and variable covariances” suggests a complex model with freely estimated variances and covariances, which could have implications for the number of free parameters and stability. Please explain briefly why this parameterization was chosen over more constrained models.

We clarified this aspect of our analyses with the following text, now located in the Statistical Analyses section:

"Models were estimated with free parameters for both variances and covariances across all profiles."

The above sentence is also footnoted with: "Model convergence issues were encountered when additional parameter constraints on the covariance structure were imposed for some of the LPA models."

4) ANOVA should be presented in a Table, with additional information about effect sizes and post hoc comparisons.

We have added a new Table 3 with the above information, as well as a new Figure 2 that illustrates the differences between levels of zPSMU between the profiles.

5) Given the wide age range of the sample, it should be considered whether age and sex/gender should be examined as covariates or profile predictors.

Age and sex similarities or differences were not a focus of the study, and we did not make any predictions regarding these variables. Nonetheless, given the reviewer's suggestion above, we have now included additional analyses and results that address their suggestions at the end of the results section and briefly address the results in the Discussion.

Discussion

1) Again the chapter begins by immediately presenting the results, while a brief reminder of the study’s aims could be helpful here.

We have edited the manuscript as noted above.

2) Line 420-422: (We are not suggesting that our data provide any evidence of causal relations between anxiety or avoidance and GN, VN, and IC.) This sentence should be permeating the whole spirit of the Discussion writing (and not stated in a parenthesis), given that the study is clearly correlational.

We have edited the manuscript as noted above.

3) Discussion about the neutral profile should be more explicit since the sample was recruited online and could be prone to careless or patterned responding. For this, you could examine whether the neutral profile differs from other profiles on the grounds of the way respondents answered (i.e. response quality, recruitment source, completion time, attention checks etc). If the neutral profile is potentially an artefact, then its substantive interpretation should be cautious. The authors might also test whether the main conclusions remain when the neutral profile is excluded.

Our revised LPA results no longer include the original neutral profile. We are grateful for the suggestions of the reviewers, which led us to recompute the LPA, and led to a four-profile solution that eliminated the neutral and grandiose profiles.

4) Maybe consider moving the Conclusions section at the closing part of the manuscript.

We have moved the Conclusions to the end of the text, following Limitations and future research.

Minor corrections:

-Line 211: for which there we no missing data on scale items
- Line 379: The manuscript reports significant p values such as “less than .000.” This should be revised to “p < .001.”

We corrected the above mistakes.

Good luck with your work!

Sincere thanks to the above three reviewers. Their keen reading of the original manuscript, and thorough suggestions, has led us to revisit the analyses and revise the manuscript in a manner that we hope provides a more clear and compelling case for the value of the study.
