# Supplementary material for: Attachment, grandiose and vulnerable narcissism, interpersonal competence, and problematic social media use: A latent profile analysis
Source: PLoS One. 2026 Oct 21;21(10):e0359569. doi: 10.1371/journal.pone.0359569 (PMC13686506; doi:10.1371/journal.pone.0359569)
Supplement: Supplementary file 2 [file pone.0359569.s003.docx]

We thank the reviewers again for their thorough examination of our revised manuscript. Their suggestions have led us to revise the manuscript in ways that strengthen the results and text, and we hope will benefit readers.

Below are comments and suggestions from Reviewers, followed by our indented replies to each suggestion.

======================================

6. Review Comments to the Author

Reviewer #1: I would like to thank the authors for the effort they have put into addressing the comments made by all the reviewers. I believe the discussion has substantially improved the article and its presentation. As far as I am concerned, I believe the suggested changes have been incorporated, and where they have not been implemented, this has been properly justified. Congratulations.

Reviewer #2: Thank you for having considered by comments and alterations. The manuscript has been considerably improved and the study seems to tell an interesting and comprehensive "story". The introduction has been more focused and alot of issues have been elaborated enhancing coherence and meaning making in terms of the links among the different sections of the study.

We are grateful to Reviewers #1 and #2 for their expertise and very helpful suggestions regarding our work.

Reviewer #3: Dear Author(s), thank you for re-submitting the manuscript Attachment, Grandiose and Vulnerable Narcissism, Interpersonal Competence, and Problematic Social Media Use: A Latent Profile Analysis. The revision is substantially responsive to the initial comments, and the manuscript has improved greatly into a more coherent person-centered paper. The authors now clearly state that the variable-centered analyses are secondary and that the main contribution lies in identifying attachment-related latent profiles and examining their differences in problematic social media use. However, some remaining technical and presentation issues need to be corrected for the paper to be fully publishable.

We are grateful for Reviewer #3's expertise, as well as the suggestions below.

Below you can find recommendations for improvement:
- The authors have substantially improved the treatment of the PSMU measure, describing a split-half validation approach, including EFA in one half, CFA in the other half, CFA in the full sample, KMO, Bartlett’s test, AVE, α, and ω. Although this does not fully solve the lack of external validation, it meaningfully strengthens the manuscript. That said, the authors should still acknowledge that the measure was not externally validated against an established measure such as the Bergen Social Media Addiction Scale or Social Media Disorder Scale.

We have added this acknowledgement in the 'Limitations and future research' section.

The CFA RMSEA values are also relatively high (.10 in the split-half CFA and .09 in the full-sample CFA), so describing the fit as simply “good” may be too strong. A more cautious phrasing would be preferable.

We have revised our manuscript to describe our model fit results as 'acceptable' rather than 'good'.

- The manuscript states that “lower values” indicate better log-likelihood, BIC, SABIC, and AIC. This is correct for AIC/BIC/SABIC, but not for log-likelihood as higher/less negative log-likelihood values indicate better fit. The authors should revise this sentence.

We have corrected the text in the Statistical analyses section and the Results section.

- Check again the sample items for attachment anxiety and attachment avoidance as they seem to be reversed. “I don’t feel comfortable opening up to romantic partners” reflects avoidance, whereas “I worry about being abandoned” reflects anxiety. This should be corrected because Table 1 was added specifically to improve clarity.

Thank you kindly. We have corrected this error in Table 1.

- The Discussion is much improved, but the practical implications could be stated more directly: for example, how identifying fearful versus anxious profiles may help researchers, clinicians, or prevention programs better understand risk for problematic social media use.

We have added a second-to-last paragraph in the discussion addressing the practical implications as noted above.

- Several minor typographical and wording errors need attention, (e.g. “non-signficant,” “the the ECR-12,” “alinged,” “instrusions,” and “participants who with.”)
Good luck with your work!

Thank you kindly. We have corrected the errors noted above.

Reviewer #4: Thank you for the revised version of this paper.

After reading it, I can say that this manuscript has improved clearly on the basis of the p`revious comments provided, and I think the main concerns raised in the previous round have been addressed satisfactorily. The revised analyses are more coherent, the four-profile solution is easier to justify theoretically, and the manuscript now reads much more clearly in terms of its primary objective and overall structure. In my view, the paper is now very close to being acceptable, with only a few minor points that would benefit from a final round of polishing.

My remaining comments are minor and mostly editorial:

Page 14, lines 244-5: please correct “for which there we no missing data on scale items.” This appears to be a simple wording error and should read “were no missing data.”

We corrected this error.

Page 19, lines 379-80: please revise “the 4-profile solution (a) was the highest profile after which the model became non-significant for the first time…”. The wording is slightly awkward and could be made more direct.

We edited this text as noted.

Page 22, lines 420-1: the sentence reporting the post-hoc comparisons is clear, but I would standardize the p-value style throughout the manuscript. In particular, expressions such as “all p ≤ .023” should follow the same formatting conventions used elsewhere in the paper.

We removed “all p ≤ .023” and point the reader to specific comparisons and *p* values in Table 4, rather than reiterate all six sets of results in the paragraph text.

Page 24, lines 457-62: the transition into the variable-centered results is still a bit long. Since the person-centered component is now the main contribution, this opening sentence or two could be tightened slightly so the discussion reaches the profile interpretation more quickly.

We edited the text to eliminate unnecessary wording.

Page 30, lines 628-42: the conclusions are now much better aligned with the revised profile solution, but I would still suggest one final check to ensure that all references to the earlier six-profile version have been fully removed from the manuscript and tracked-changes file.

We have carefully reviewed the Discussion section as suggested.

Overall, I think the manuscript is in good shape and would support it after these minor adjustments.
Thanks for your contribution.

Our very sincere thanks to all reviewers.
