# Supplementary material for: Attachment, grandiose and vulnerable narcissism, interpersonal competence, and problematic social media use: A latent profile analysis
Source: PLoS One. 2026 Oct 21;21(10):e0359569. doi: 10.1371/journal.pone.0359569 (PMC13686506; doi:10.1371/journal.pone.0359569)
Supplement: Supplementary file 3 [file pone.0359569.s004.docx]

We thank the reviewers again for their thorough examination of our revised manuscript. Their suggestions have led us to further revise and improve the manuscript, which we hope will benefit readers.

Below are comments and suggestions from Reviewers, followed by our indented replies to each suggestion.

======================================

Reviewer #3: Dear Author(s), thank you for the careful revision. The manuscript has improved substantially, and the authors have adequately addressed most of the substantive concerns raised in the previous round. The four-profile solution is now presented more clearly, the log-likelihood interpretation and attachment sample items have been corrected, the absence of external validation of the PSMU measure is acknowledged, and references to the earlier six-profile solution appear to have been removed.

A small number of issues remain before the manuscript is ready for acceptance:
1. On p. 15, lines 287–291, the CFA results are described as demonstrating “overall acceptable model fit.” Because RMSEA was .10 in the holdout sample and .09 in the full sample—both above the authors’ stated criterion of ≤ .08—the results would be more accurately described as showing mixed fit: acceptable according to CFI, TLI, and SRMR, but weaker fit according to RMSEA.

Thank you. We have made the above change to the text on page 15.

2. The practical-implications paragraph on pp. 29–30, lines 586–593 addresses the previous revision’s comment, but its wording should be revisited. In particular, statements that avoidant attachment “does not predict” high PSMU and that individuals are “at greater risk of failing to develop necessary interpersonal skills” imply causal or developmental conclusions that cannot be supported by this cross-sectional study.

Thank you. We have edited the language on pages 29-30 to remove any implication of causal or developmental conclusions. We also noticed and edited text in the first Discussion paragraph (p. 23) for the same purpose (e.g., we replaced "develop" with "express" on line 454, Track Changes MS).

The sentence beginning “Yet, of practical use to researchers…” also requires grammatical revision.

Thank you. We have edited the referenced sentence.

3. The opening of the Discussion remains somewhat repetitive. The sentences “We interpret these findings in greater detail below” and “Below we briefly interpret…” could be combined so that the discussion moves more directly to the findings. Also, I suggest that you omit the reference to the H8 and H9 hypothesis at the beginning of the chapter, as it reads confusing. Leave only the sentence without the two brackets. You can mention them in the right order afterwards.

Thank you. We have made the above corrections.

4. A final proofread is required. Remaining errors include, for example, “alinged” (p. 21, line 404), “naricissistic” (p. 27, line 523), “signficant” (p. 32, line 640), “handing” rather than “handling” in Table 1.

Thank you. We have made the above corrections. We also noticed and corrected a grammatical error in the Abstract (added 'the' on line 40), and a wording error in the section Limitations and future research (line 627 Track Changes MS, changed 'Fourth' to 'Third').

5. In Table 3, “Bold indicates best fitted model” would be more accurate as “Bold indicates the selected model,” because different indices favour different solutions and the four-profile model was selected through a combination of statistical and theoretical considerations.

Thank you. We have made the above corrections.

After these minor corrections I believe the manuscript could be suitable for publication.

We are sincerely grateful to all reviewers for their thorough and expert reviews of our manuscript.
