# Supplementary material for: Attachment, grandiose and vulnerable narcissism, interpersonal competence, and problematic social media use: A latent profile analysis
Source: PLoS One. 2026 Oct 21;21(10):e0359569. doi: 10.1371/journal.pone.0359569 (PMC13686506; doi:10.1371/journal.pone.0359569)
Supplement: Peer review history

# Peer review: Decision Letter 0

**DOI:** 10.1371/journal.pone.0359569.r001

**Authors:** Sergio A. Useche

10 May 2026

Dear Dr. Bjornsen,

Thank you for submitting your manuscript to PLOS ONE. After careful consideration, we feel that it has merit but does not fully meet PLOS ONE’s publication criteria as it currently stands. Therefore, we invite you to submit a revised version of the manuscript that addresses the points raised during the review process.

plosone@plos.org. When you're ready to submit your revision, log on to https://www.editorialmanager.com/pone/ and select the 'Submissions Needing Revision' folder to locate your manuscript file.

- A letter that responds to each point raised by the academic editor and reviewer(s). You should upload this letter as a separate file labeled 'Response to Reviewers'.
- A marked-up copy of your manuscript that highlights changes made to the original version. You should upload this as a separate file labeled 'Revised Manuscript with Track Changes'.
- An unmarked version of your revised paper without tracked changes. You should upload this as a separate file labeled 'Manuscript'.

If you would like to make changes to your financial disclosure, please include your updated statement in your cover letter. Guidelines for resubmitting your figure files are available below the reviewer comments at the end of this letter.

If applicable, we recommend that you deposit your laboratory protocols in protocols.io to enhance the reproducibility of your results. Protocols.io assigns your protocol its own identifier (DOI) so that it can be cited independently in the future. For instructions see: https://journals.plos.org/plosone/s/submission-guidelines#loc-laboratory-protocols. Additionally, PLOS ONE offers an option for publishing peer-reviewed Lab Protocol articles, which describe protocols hosted on protocols.io. Read more information on sharing protocols at https://plos.org/protocols?utm_medium=editorial-email&utm_source=authorletters&utm_campaign=protocols.

As the corresponding author, your ORCID iD is verified in the submission system and will appear in the published article. PLOS supports the use of ORCID, and we encourage all coauthors to register for an ORCID iD and use it as well. Please encourage your coauthors to verify their ORCID iD within the submission system before final acceptance, as unverified ORCID iDs will not appear in the published article. *Only*  the individual author can complete the verification step; PLOS staff *cannot*  verify ORCID iDs on behalf of authors.

We look forward to receiving your revised manuscript.

Kind regards,

Sergio A. Useche, Ph.D.

Academic Editor

PLOS One

Journal Requirements:

When submitting your revision, we need you to address these additional requirements.

1. Please ensure that your manuscript meets PLOS ONE's style requirements, including those for file naming. The PLOS ONE style templates can be found at

https://journals.plos.org/plosone/s/file?id=wjVg/PLOSOne_formatting_sample_main_body.pdf and https://journals.plos.org/plosone/s/file?id=ba62/PLOSOne_formatting_sample_title_authors_affiliations.pdf

2. Please amend your authorship list in your manuscript file to include author Chris Bjornsen.

3. Please amend the manuscript submission data (via Edit Submission) to include author Chris A. Bjornsen.

4. If the reviewer comments include a recommendation to cite specific previously published works, please review and evaluate these publications to determine whether they are relevant and should be cited. There is no requirement to cite these works unless the editor has indicated otherwise.

[Note: HTML markup is below. Please do not edit.]

Reviewers' comments:

Reviewer's Responses to Questions

**Comments to the Author**

1. Is the manuscript technically sound, and do the data support the conclusions?

Reviewer #1: Yes

Reviewer #2: Yes

Reviewer #3: Yes

**********

2. Has the statistical analysis been performed appropriately and rigorously?

Reviewer #1: Yes

Reviewer #2: Yes

Reviewer #3: Yes

**********

3. Have the authors made all data underlying the findings in their manuscript fully available?

The PLOS Data policy

Reviewer #1: Yes

Reviewer #2: Yes

Reviewer #3: Yes

**********

4. Is the manuscript presented in an intelligible fashion and written in standard English?

Reviewer #1: Yes

Reviewer #2: Yes

Reviewer #3: Yes

**********

Reviewer #1: I would like to begin by highlighting several strengths of the manuscript in relation to the journal’s publication criteria. The study presents original empirical research that integrates theoretically relevant constructs often examined separately (attachment, grandiose and vulnerable narcissism, interpersonal competence, and problematic social media use). The analytical strategy is clearly described and combines variable-centered and person-centered approaches through correlational analyses and latent profile analysis. The sample size is adequate for the statistical procedures conducted, and model selection criteria and fit indices are reported transparently, allowing the reader to evaluate the analytical decisions. In addition, the availability of data, materials, and code in an open repository substantially enhances transparency and reproducibility, aligning with current standards for research integrity and data availability.

However, I would like to highlight several general issues that should be addressed before the manuscript can be considered suitable for publication.

First, although the hypotheses (H1–H9) are clearly stated, the overall analytical architecture lacks coherence. The manuscript combines variable-centered hypotheses, person-centered predictions through LPA, and a subsequent validation step examining differences in PSMU across profiles. While using both approaches is acceptable, the manuscript does not clarify how they relate to one another or which constitutes the primary objective of the study. It remains uncertain whether the central contribution lies in confirming established associations, identifying latent subgroups, or explaining variation in problematic social media use. As presented, the transition from correlational analyses to profile modeling and validation reads as sequential rather than theoretically driven. Clarifying the hierarchy of objectives and organizing the hypotheses accordingly would strengthen the internal logic of the study.

Second, the measurement of PSMU is a vulnerable aspect of the manuscript. The instrument was developed ad hoc based on DSM-5 criteria for alcohol use disorder and is not a previously validated measure of problematic social media use. After conducting an exploratory factor analysis, four items were removed and only six were retained, all within the same sample used for the main analyses. No cross-validation or confirmatory factor analysis was conducted to verify the retained structure, and no evidence of convergent, discriminant, or criterion validity is provided. The manuscript also does not compare this measure with established instruments such as the Bergen Social Media Addiction Scale or the Social Media Disorder Scale, both validated in U.S. populations. This limits the interpretability of the findings and makes it difficult to situate the results within the existing literature. Because PSMU is used to validate the latent profile solution, greater attention to its measurement is necessary. This limitation should be clearly acknowledged.

Third, concerns also arise regarding item removal across several measures. One item was removed from the SD3, two from the HNS, and four from the PSMU scale, all within the same dataset subsequently used for the primary analyses. Removing items without cross-validation increases the likelihood that model fit improvements reflect sample-specific characteristics rather than stable structural properties. For validated instruments such as the SD3 and HNS, modifying the original structure requires stronger theoretical justification and discussion of the implications for construct coverage and comparability with prior research. As noted above, the absence of independent validation limits confidence in the stability of the revised scales. Clearer justification and acknowledgment of this limitation would strengthen the manuscript.

Specific comments

The abstract should state more clearly the primary objective of the study and the main conclusions derived from it.

The introduction is overly extensive and at times more descriptive than integrative. Although the literature review is thorough, the detailed presentation of numerous associations makes it difficult to identify the central focus of the study. A more selective synthesis emphasizing the most consistent findings would improve clarity. The introduction would also benefit from a concluding paragraph that explicitly states the primary objective before presenting the hypotheses.

Given the number of constructs involved (attachment, grandiose narcissism, vulnerable narcissism, interpersonal competence, and PSMU), including a concise table summarizing their conceptual definitions and operationalizations would improve readability.

Consistency in abbreviations should be reviewed. CFA is introduced on page 10, yet the full term is later repeated alongside the abbreviation. Some scales are properly introduced with their full name followed by the abbreviation, while others, such as ICQ-15, appear without prior clarification. Standardizing abbreviations would improve clarity.

The use of italics should also be revised. In some instances, italics appear to be used for emphasis without a clear rationale, such as on page 23, line 489, where the words specifically and also are italicized. Formatting should be consistent and limited to standard academic conventions.

The format of Table 1 should be aligned with the journal’s presentation guidelines.

Finally, the discussion would benefit from a clearer structure. It would be helpful to restate the primary objective before interpreting the findings and to organize the discussion around the stated hypotheses, indicating which were supported. A more structured discussion would improve coherence between the introduction, hypotheses, and results.

Reviewer #2: The study is well-written and interesting. However, I have some comments for authors to reconsider before publication.

HYPOTHESES

p.9. Hypotheses 8 should be shortened

The sentence “Our results generally supported the hypotheses. “ could be omitted since the hypotheses will be tested in the results section and will be discussed in the next section- Discussion

In the Discussion sections authors should do a lot of work. An introductory paragraph should present the aim of the study and a summary of the findings. Then, start the discussion with the most important findings which depict the contribution of the study to the relevant literature.

On page 20 the information in the brackets should appear in the main text. The same throughout the manuscript.

My suggestion is the Discussion to start with the profiles with regard to the use of SM and incorporate where appropriate the information before the Latent Analysis, in the current version.

Practical implications should be of interest.

Reviewer #3: Dear Authors, thank you for submitting the manuscript “Attachment, Grandiose and Vulnerable Narcissism, Interpersonal Competence, and Problematic Social Media Use: A Latent Profile Analysis”. The article falls into the scope of the journal, as it examines the associations between the two dimensions of attachment (anxiety and avoidance), grandiose and vulnerable narcissism, interpersonal competence, and problematic social media use among. Please find below some suggestions listed from the most important by sections:

Abstract

Please start the abstract with one sentence stating the present state of the subject presented and why it is important to investigate it further (in line 31). Also you could state firmly that it concerns a cross-sectional study, based on a self-reported survey.

Introduction

1) First, at the beginning of the literature review, it feels that the text opens rather immediately to the dimensions and research underscoring attachment, while some information about the concept itself is missing from the classical studies (i.e. a widely accepted definition/description of attachment). Furthermore, it would also be helpful to define shortly attachment dimensions, as well.

2) The hypotheses section is detailed but could be reorganized. H1–H7 are correlational, whereas H8–H9 are person-centered and auxiliary-outcome hypotheses. Separating these into “variable-centered hypotheses” and “person-centered hypotheses” would improve clarity.

3) Line 200: Avoid discussing the results in the Introduction before presenting all evidence of your methodology, data and results.

Method

1) The most important concern concerns the PSMU measure. The authors used 10 original items based largely on DSM-5 alcohol use disorder criteria, conducted an EFA, retained six items from one factor, and then used this six-item score as the outcome/auxiliary variable. This decision needs more careful justification. The construct of problematic social media use is well established, and several validated measures are available. The use of a newly created, shortened, post hoc selected measure raises concerns about construct validity.

2) Concerning the instruments, further evidence of CFA are required. Maybe also consider reporting information about data suitability for CFA, together with (AVE) and inter-factor correlations to evaluate convergent validity and the distinctiveness of the factors. Finally, you could report both McDonald’s omega and Cronbach’s alpha for internal consistency.

3) The grandiose narcissism scale was taken from the SD3, and one item was removed after CFA. The vulnerable narcissism scale was based on the HNS, and two items were removed after CFA. These item removals should be better justified. You should explain whether the decisions were theory-driven or purely statistical, whether modification indices guided them, and whether the resulting scales remain comparable to prior research.

4) You also need to use the same level of interpretation of reliability indices (e.g. line 226, it is stated as “good” (a = .90), but in line 252 a=.88 is interpreted as “excellent”. Consider reporting the cut-off scores in the Data Analysis section.

5) It would be helpful to report the versions of the statistical platforms that you used (i.e. R and Jamovi)

6) In the Statistical Analysis you go to lengths to describe the LPA (which is very helpful and commendable), but information about EFA, CFA and correlations are missing (e.g. cut-offs, model fit assumptions). Also, how you treated possible outliers.

Results

1) The text about the correlation results repeats Table data, which makes it redundant. You could highlight the most interesting findings, instead, referring to the Table for indices inspection.

2) In lines 320-321 it is mentioned: “The LPA was computed using the TidyLPA package in R [95] with the EM algorithm estimation protocol.” This could be more fittingly be moved into the Statistical Analyses section and leave only the results in the present section.

3) Furthermore, it should also be clarified how the exact TidyLPA model is specified. The statement that models “accounted for variable variances and variable covariances” suggests a complex model with freely estimated variances and covariances, which could have implications for the number of free parameters and stability. Please explain briefly why this parameterization was chosen over more constrained models.

4) ANOVA should be presented in a Table, with additional information about effect sizes and post hoc comparisons.

5) Given the wide age range of the sample, it should be considered whether age and sex/gender should be examined as covariates or profile predictors.

Discussion

1) Again the chapter begins by immediately presenting the results, while a brief reminder of the study’s aims could be helpful here.

2) Line 420-422: (We are not suggesting that our data provide any evidence of causal relations between anxiety or avoidance and GN, VN, and IC.) This sentence should be permeating the whole spirit of the Discussion writing (and not stated in a parenthesis), given that the study is clearly correlational.

3) Discussion about the neutral profile should be more explicit since the sample was recruited online and could be prone to careless or patterned responding. For this, you could examine whether the neutral profile differs from other profiles on the grounds of the way respondents answered (i.e. response quality, recruitment source, completion time, attention checks etc). If the neutral profile is potentially an artefact, then its substantive interpretation should be cautious. The authors might also test whether the main conclusions remain when the neutral profile is excluded.

4) Maybe consider moving the Conclusions section at the closing part of the manuscript.

Minor corrections:

-Line 211: for which there we no missing data on scale items

- Line 379: The manuscript reports significant p values such as “less than .000.” This should be revised to “p < .001.”

Good luck with your work!

**********

what does this mean?). If published, this will include your full peer review and any attached files.

If you choose “no”, your identity will remain anonymous but your review may still be made public.

**Do you want your identity to be public for this peer review?**  For information about this choice, including consent withdrawal, please see our Privacy Policy

Reviewer #1: **Yes:**  Alfredo Zarco Alpuente

Reviewer #2: No

Reviewer #3: **Yes:**  Panayiotis G. Lianos

**********

[NOTE: If reviewer comments were submitted as an attachment file, they will be attached to this email and accessible via the submission site. Please log into your account, locate the manuscript record, and check for the action link "View Attachments". If this link does not appear, there are no attachment files.]

To ensure your figures meet our technical requirements, please review our figure guidelines: https://journals.plos.org/plosone/s/figures

You may also use PLOS’s free figure tool, NAAS, to help you prepare publication quality figures: https://journals.plos.org/plosone/s/figures#loc-tools-for-figure-preparation.

NAAS will assess whether your figures meet our technical requirements by comparing each figure against our figure specifications.

# Author comment: Author response to Decision Letter 1

**DOI:** 10.1371/journal.pone.0359569.r002

4 Jun 2026

We thank the reviewers for their thorough examination of our original manuscript. Their suggestions have indeed led us to revise the manuscript in ways that strengthen the results and text, and we hope will benefit readers.

Below are comments and suggestions from Reviewers 1 - 3, followed by our indented replies to each suggestion.

======================================

Reviewer #1: I would like to begin by highlighting several strengths of the manuscript in relation to the journal’s publication criteria. The study presents original empirical research that integrates theoretically relevant constructs often examined separately (attachment, grandiose and vulnerable narcissism, interpersonal competence, and problematic social media use). The analytical strategy is clearly described and combines variable-centered and person-centered approaches through correlational analyses and latent profile analysis. The sample size is adequate for the statistical procedures conducted, and model selection criteria and fit indices are reported transparently, allowing the reader to evaluate the analytical decisions. In addition, the availability of data, materials, and code in an open repository substantially enhances transparency and reproducibility, aligning with current standards for research integrity and data availability.

However, I would like to highlight several general issues that should be addressed before the manuscript can be considered suitable for publication.

First, although the hypotheses (H1–H9) are clearly stated, the overall analytical architecture lacks coherence. The manuscript combines variable-centered hypotheses, person-centered predictions through LPA, and a subsequent validation step examining differences in PSMU across profiles. While using both approaches is acceptable, the manuscript does not clarify how they relate to one another or which constitutes the primary objective of the study. It remains uncertain whether the central contribution lies in confirming established associations, identifying latent subgroups, or explaining variation in problematic social media use. As presented, the transition from correlational analyses to profile modeling and validation reads as sequential rather than theoretically driven. Clarifying the hierarchy of objectives and organizing the hypotheses accordingly would strengthen the internal logic of the study.

We have added a new opening paragraph in the Introduction that is designed to clarify the primary objectives of the study and explains that the variable-centered correlational analyses are of secondary importance and are primarily meant to provide support and context for the person-centered latent profile analysis and subsequent examination of profile differences in PSMU.

Second, the measurement of PSMU is a vulnerable aspect of the manuscript. The instrument was developed ad hoc based on DSM-5 criteria for alcohol use disorder and is not a previously validated measure of problematic social media use. After conducting an exploratory factor analysis, four items were removed and only six were retained, all within the same sample used for the main analyses. No cross-validation or confirmatory factor analysis was conducted to verify the retained structure, and no evidence of convergent, discriminant, or criterion validity is provided. The manuscript also does not compare this measure with established instruments such as the Bergen Social Media Addiction Scale or the Social Media Disorder Scale, both validated in U.S. populations. This limits the interpretability of the findings and makes it difficult to situate the results within the existing literature. Because PSMU is used to validate the latent profile solution, greater attention to its measurement is necessary. This limitation should be clearly acknowledged.

We addressed the above concern in our revision using cross-validation, explained in detail in the revised manuscript. We randomly split the sample and computed an exploratory factor analysis (EFA) on the first half. The EFA produced a factor containing the same six-items used in the original manuscript to create the PSMU scale. We then computed a confirmatory factor analysis (CFA) on the second half of the split file using the six items identified by the EFA. This CFA confirmed the factor structure of the EFA. Finally, we computed a CFA on the full sample using the identified six items, confirming the factor structure of the PSMU scale for the full sample. The EFA and CFAs demonstrated overall good model fits.

Regarding the use of an original set of items, rather than the Bergen Social Media Addiction Scale (BSMS), we submit that the original items and those retained in our PSMU scale are qualitatively quite similar to the BSMS items. More specifically, our PSMU items address characteristics of addiction (in this case, social media addiction, more commonly labeled problematic social media use in recent studies) that are also addressed by the items in the BSMS, namely relapse/loss of control, conflict/functional impairment, mood modification, and craving/tolerance.

Also, similar to Andreassen et al's. (2016) six-item scale, which was significantly correlated with anxiety (r = .34, p < .01), evidence of convergent validity of our six-item PSMU scale is reflected in the significant positive correlations between the PSMU and attachment anxiety (r = .49, p < .001) and vulnerable narcissism (r = .42, p < .001). Evidence of discriminant validity is reflected in the significant negative correlation between PSMU and interpersonal competence (r = -.18, p < .001).

Third, concerns also arise regarding item removal across several measures. One item was removed from the SD3, two from the HNS, and four from the PSMU scale, all within the same dataset subsequently used for the primary analyses. Removing items without cross-validation increases the likelihood that model fit improvements reflect sample-specific characteristics rather than stable structural properties. For validated instruments such as the SD3 and HNS, modifying the original structure requires stronger theoretical justification and discussion of the implications for construct coverage and comparability with prior research. As noted above, the absence of independent validation limits confidence in the stability of the revised scales. Clearer justification and acknowledgment of this limitation would strengthen the manuscript.

We originally considered our confirmatory approach (CFA) to demonstrate the psychometric properties in our study of previously published scales (for attachment anxiety and avoidance, grandiose and vulnerable narcissism, and interpersonal competence) to be a more stringent approach than relying solely on common measures of reliability, such as Cronbach's alpha. However, the critiques of reviewers regarding dropping scale items are reasonable. Thus, we reverted to the more common approach of computing and reporting Cronbach and McDonald's reliability coefficients for established scales. (We explained how we addressed the reviewer's concern regarding the PSMU scale above.)

After computing full-scale scores for grandiose narcissism and vulnerable narcissism, using all items in those published measures, we computed reliability coefficients for all scales and include them in the Measures section of our revised manuscript. We also re-ran all analyses, which resulted in an LPA model that indeed more strongly supports our original hypothesis (H8), predicting profiles reflecting the four traditional attachment styles. The revised LPA supports a four-profile structure, and the profiles reflect levels of attachment anxiety and attachment avoidance that characterize the four traditional attachment styles (secure, avoidant, anxious, and fearful). The profiles are also characterized by levels of grandiose narcissism, vulnerable narcissism, and interpersonal competence that largely support our predictions regarding relations between attachment styles and narcissism and interpersonal competence. Lastly, the validation of the profiles using ANOVA and post-hoc analyses to compare levels of (z-transformed) PSMU among the four LPA profiles demonstrated statistically significant differences between all four profiles, also supporting our original hypothesis (H9). The levels of PSMU across the four profiles also support and strengthen findings of previous studies indicating that PSMU is lower among securely attached adults, higher for avoidantly attached adults, and higher still for anxiously attached adults. Our results also extend previous literature by demonstrating PSMU is highest for adults with high levels of both anxious and avoidant attachment, which is the pattern that characterizes the fearful attachment style.

Specific comments

The abstract should state more clearly the primary objective of the study and the main conclusions derived from it.

We have revised the abstract as suggested.

The introduction is overly extensive and at times more descriptive than integrative. Although the literature review is thorough, the detailed presentation of numerous associations makes it difficult to identify the central focus of the study. A more selective synthesis emphasizing the most consistent findings would improve clarity. The introduction would also benefit from a concluding paragraph that explicitly states the primary objective before presenting the hypotheses.

We appreciate the reviewer's concern regarding the introduction. We have added a paragraph at the beginning of the introduction (at the suggestion of the present reviewer, above), as well as a concluding paragraph at the end of the introduction, that we believe integrate the separate sections of the introduction. Given the inherent complexity of the LPA, we believe that a concise but pointed examination of the associations between the different variables (three of which are only one paragraph in length) is useful. We spent considerable time identifying existing studies on each of the associations between the variables specifically to focus each section on the best available evidence. We hope that given the other structural / explanatory changes we have made to the manuscript, prompted by the expert suggestions of the reviewers, the introduction no longer seems as descriptive as in the original submission, and instead communicates a useful integration of the existing literature.

Given the number of constructs involved (attachment, grandiose narcissism, vulnerable narcissism, interpersonal competence, and PSMU), including a concise table summarizing their conceptual definitions and operationalizations would improve readability.

We have added a new Table 1 to the end of the Introduction summarizing the variables/constructs.

Consistency in abbreviations should be reviewed. CFA is introduced on page 10, yet the full term is later repeated alongside the abbreviation. Some scales are properly introduced with their full name followed by the abbreviation, while others, such as ICQ-15, appear without prior clarification. Standardizing abbreviations would improve clarity.

We have made the suggested corrections throughout the manuscript.

The use of italics should also be revised. In some instances, italics appear to be used for emphasis without a clear rationale, such as on page 23, line 489, where the words specifically and also are italicized. Formatting should be consistent and limited to standard academic conventions.

We have removed italics except for scale anchors.

The format of Table 1 should be aligned with the journal’s presentation guidelines.

We have revised the format of our Tables accordingly.

Finally, the discussion would benefit from a clearer structure. It would be helpful to restate the primary objective before interpreting the findings and to organize the discussion around the stated hypotheses, indicating which were supported. A more structured discussion would improve coherence between the introduction, hypotheses, and results.

We have revised the discussion as suggested above.

Reviewer #2: The study is well-written and interesting. However, I have some comments for authors to reconsider before publication.

HYPOTHESES

p.9. Hypotheses 8 should be shortened

We shortened H8 as suggested.

The sentence “Our results generally supported the hypotheses. “ could be omitted since the hypotheses will be tested in the results section and will be discussed in the next section-

We omitted the sentence noted above.

Discussion

In the Discussion sections authors should do a lot of work. An introductory paragraph should present the aim of the study and a summary of the findings. Then, start the discussion with the most important findings which depict the contribution of the study to the relevant literature.

We have revised the Discussion as suggested above.

On page 20 the information in the brackets should appear in the main text. The same throughout the manuscript.

We have corrected the manuscript as noted above.

My suggestion is the Discussion to start with the profiles with regard to the use of SM and incorporate where appropriate the information before the Latent Analysis, in the current version.

Practical implications should be of interest.

We have edited the manuscript as noted above.

Reviewer #3: Dear Authors, thank you for submitting the manuscript “Attachment, Grandiose and Vulnerable Narcissism, Interpersonal Competence, and Problematic Social Media Use: A Latent Profile Analysis”. The article falls into the scope of the journal, as it examines the associations between the two dimensions of attachment (anxiety and avoidance), grandiose and vulnerable narcissism, interpersonal competence, and problematic social media use among. Please find below some suggestions listed from the most important by sections:

Abstract

Please start the abstract with one sentence stating the present state of the subject presented and why it is important to investigate it further (in line 31). Also you could state firmly that it concerns a cross-sectional study, based on a self-reported survey.

We edited the abstract as suggested above.

Introduction

1) First, at the beginning of the literature review, it feels that the text opens rather immediately to the dimensions and research underscoring attachment, while some information about the concept itself is missing from the classical studies (i.e. a widely accepted definition/description of attachment). Furthermore, it would also be helpful to define shortly attachment dimensions, as well.

We added information as suggested above at the beginning of the Introduction.

2) The hypotheses section is detailed but could be reorganized. H1–H7 are correlational, whereas H8–H9 are person-centered and auxiliary-outcome hypotheses. Separating these into “variable-centered hypotheses” and “person-centered hypotheses” would improve clarity.

We edited the text at the beginning of the Hypotheses section to identify variable-centered vs person-centered hypotheses.

3) Line 200: Avoid discussing the results in the Introduction before presenting all evidence of your methodology, data and results.

We omitted the sentence as noted above.

Method

1) The most important concern concerns the PSMU measure. The authors used 10 original items based largely on DSM-5 alcohol use disorder criteria, conducted an EFA, retained six items from one factor, and then used this six-item score as the outcome/auxiliary variable. This decision needs more careful justification. The construct of problematic social media use is well established, and several validated measures are available. The use of a newly created, shortened, post hoc selected measure raises concerns about construct validity.

We addressed the above concern in our revision using cross-validation, explained in detail in the revised manuscript. We randomly split the sample and computed an exploratory factor analysis (EFA) on the first half. The EFA produced a factor containing the same six-items used in the original manuscript to create the PSMU scale. We then computed a confirmatory factor analysis (CFA) on the second half of the split file using the six items identified by the EFA. This CFA confirmed the factor structure of the EFA. Finally, we computed a CFA on the full sample using the identified six items, confirming the factor structure of the PSMU scale for the full sample. The EFA and CFAs demonstrated overall good model fits.

Regarding the use of an original set of items, rather than the Bergen Social Media Addiction Scale (BSMS), we submit that the original items and those retained in our PSMU scale are qualitatively quite similar to the BSMS items. More specifically, our PSMU items address characteristics of addiction (in this case, social media addiction, more commonly labeled problematic social media use in recent studies) that are also addressed by the items in the BSMS, namely relapse/loss of control, conflict/functional impairment, mood modification, and craving/tolerance.

Also, similar to Andreassen et al's. (2016) six-item scale, which was significantly correlated with anxiety (r = .34, p < .01), evidence of convergent validity of our six-item PSMU scale is reflected in the significant positive correlations between the PSMU and attachment anxiety (r = .49, p < .001) and vulnerable narcissism (r = .42, p < .001). Evidence of discriminant validity is reflected in the significant negative correlation between PSMU and interpersonal competence (r = -.18, p < .001).

2) Concerning the instruments, further evidence of CFA are required. Maybe also consider reporting information about data suitability for CFA, together with (AVE) and inter-factor correlations to evaluate convergent validity and the distinctiveness of the factors.

We added the following information to the Statistical analyses section: "Adequacy of the PSMU scale was assessed using the Kaiser-Meyer-Olkin (KMO) measure (≥ 0.90 = excellent), Bartlett's Test of Sphericity (p ≤ .05 recommended), and Average Variance Extracted (≥ 0.50 recommended)."

Also, we added the following information to the Measures (Problematic social media use) section: "The Kaiser-Meyer-Olkin (KMO) measure verified the sampling adequacy for the EFA (KMO = 0.914), Bartlett's Test of Sphericity indicated the inter-item correlations were sufficiently large for factor analysis (χ2 (45, N = 277) = 1418, p < .001), and Average Variance Extracted (AVE, 0.54) supported the convergent validity of the items."

As constructs were analyzed as aggregate measurements (as opposed to a multi-factor measurement model), inter-factor correlations are not an issue. Furthermore, our main scale (PSMU) focused on a single factor, which we believe is supported here (and strengthened by the critique of the reviewers).

Finally, you could report both McDonald’s omega and Cronbach’s alpha for internal consistency.

We added McDonald's omega to the manuscript, in the text and Table.

3) The grandiose narcissism scale was taken from the SD3, and one item was removed after CFA. The vulnerable narcissism scale was based on the HNS, and two items were removed after CFA. These item removals should be better justified. You should explain whether the decisions were theory-driven or purely statistical, whether modification indices guided them, and whether the resulting scales remain comparable to prior research.

This issue was also raised above by Reviewer #1, and we are grateful for this critique by two reviewers, as we believe the modified results have substantially enhanced the results of our study. Our response to this issue and summary of the modifications to the manuscript are restated here:

We originally considered our confirmatory approach (CFA) to demonstrate the psychometric properties in our study of previously published scales (for attachment anxiety and avoidance, grandiose and vulnerable narcissism, and interpersonal competence) to be a more stringent approach than relying solely common measures of reliability, such as Cronbach's alpha. However, the critiques of reviewers regarding dropping scale items are understandable. Thus, we reverted to the more common approach of computing and reporting Cronbach and McDonald's reliability coefficients for established scales. (We explained how we addressed the reviewer's concern regarding the PSMU scale above.)

After computing full-scale scores for grandiose narcissism and vulnerable narcissism, using all items in those scales, we computed reliability coefficients for all scales and include them in the Measures section of our revised manuscript. We also re-ran all analyses, which resulted in an LPA model that indeed more strongly supports our original hypothesis (H8), predicting profiles reflecting the four traditional attachment styles. The revised LPA supports a four-profile structure, and the profiles reflect levels of attachment anxiety and attachment avoidance that characterize the four traditional attachment styles (secure, avoidant, anxious, and fearful). The profiles are also characterized by levels of grandiose narcissism, vulnerable narcissism, and interpersonal competence that largely support our predictions regarding relations between attachment styles and narcissism and interpersonal competence. Lastly, the validation of the profiles using ANOVA and post-hoc analyses to compare levels of (z-transformed) PSMU among the four LPA profiles demonstrated statistically significant differences between all four profiles, also supporting our original hypothesis (H9). The levels of PSMU across the four profiles also support and strengthen findings of previous studies indicating that PSMU is lower among securely attached adults, higher for avoidantly attached adults, and higher still for anxiously attached adults. Our results also extend previous literature by demonstrating PSMU is highest for adults with high levels of both anxious and avoidant attachment, which is the pattern that characterizes the fearful attachment style.

4) You also need to use the same level of interpretation of reliability indices (e.g. line 226, it is stated as “good” (a = .90), but in line 252 a=.88 is interpreted as “excellent”. Consider reporting the cut-off scores in the Data Analysis section.

We made the above corrections and additions to the manuscript.

5) It would be helpful to report the versions of the statistical platforms that you used (i.e. R and Jamovi)

We added this information to the manuscript.

6) In the Statistical Analysis you go to lengths to describe the LPA (which is very helpful and commendable), but information about EFA, CFA and correlations are missing (e.g. cut-offs, model fit assumptions). Also, how you treated possible outliers.

We added information regarding EFA and CFA model fit assumptions/cutoffs, and correlation effect sizes, to the manuscript.

An exploration of the univariate distributions flagged fewer than 3% of the data as univariate outliers on all LPA variables, which have fixed lower and upper limits and are, hence, legitimate data points. A careful examination of the outliers revealed a distinctive pattern between the vulnerable narcissism and interpersonal competence scales that justified retention of these outliers in the data set as key person-variable attributes of the data set.. Thus, we retained all cases in the analyses.

We thank the reviewers for the helpful feedback, which steered us toward a model that more clearly captured the distinctive patterns/profiles in the data set.

Results

1) The text about the correlation results repeats Table data, which makes it redundant. You could highlight the most interesting findings, instead, referring to the Table for indices inspection.

We have been careful to limit our Results text to the data that support or fail to support H1 - H7, recognizing that r and p values are also normally reported in the text.

2) In lines 320-321 it is mentioned: “The LPA was computed using the TidyLPA package in R [95] with the EM algorithm estimation protocol.” This could be more fittingly be moved into the Statistical Analyses section and leave only the results in the present section.

We moved the suggested information above and the following sentence to the Statistical Analyses section. "Models were estimated with free parameters for both variances and covariances across all profiles. "

3) Furthermore, it should also be clarified how the exact TidyLPA model is specified. The statement that models “accounted for variable variances and variable covariances” suggests a complex model with freely estimated variances and covariances, which could have implications for the number of free parameters and stability. Please explain briefly why this parameterization was chosen over more constrained models.

We clarified this aspect of our analyses with the following text, now located in the Statistical Analyses section:

"Models were estimated with free parameters for both variances and covariances across all profiles."

The above sentence is also footnoted with: "Model convergence issues were encountered when additional parameter constraints on the covariance structure were imposed for some of the LPA models."

4) ANOVA should be presented in a Table, with additional information about effect sizes and post hoc comparisons.

We have added a new Table 3 with the above information, as well as a new Figure 2 that illustrates the differences between levels of zPSMU between the profiles.

5) Given the wide age range of the sample, it should be considered whether age and sex/gender should be examined as covariates or profile predictors.

Age and sex similarities or differences were not a focus of the study, and we did not make any predictions regarding these variables. Nonetheless, given the reviewer's suggestion above, we have now included additional analyses and results that address their suggestions at the end of the results section and briefly address the results in the Discussion.

Discussion

1) Again the chapter begins by immediately presenting the results, while a brief reminder of the study’s aims could be helpful here.

We have edited the manuscript as noted above.

2) Line 420-422: (We are not suggesting that our data provide any evidence of causal relations between anxiety or avoidance and GN, VN, and IC.) This sentence should be permeating the whole spirit of the Discussion writing (and not stated in a parenthesis), given that the study is clearly correlational.

We have edited the manuscript as noted above.

3) Discussion about the neutral profile should be more explicit since the sample was recruited online and could be prone to careless or patterned responding. For this, you could examine whether the neutral profile differs from other profiles on the grounds of the way respondents answered (i.e. response quality, recruitment source, completion time, attention checks etc). If the neutral profile is potentially an artefact, then its substantive interpretation should be cautious. The authors might also test whether the main conclusions remain when the neutral profile is excluded.

Our revised LPA results no longer include the original neutral profile. We are grateful for the suggestions of the reviewers, which led us to recompute the LPA, and led to a four-profile solution that eliminated the neutral and grandiose profiles.

4) Maybe consider moving the Conclusions section at the closing part of the manuscript.

We have moved the Conclusions to the end of the text, following Limitations and future research.

Minor corrections:

-Line 211: for which there we no missing data on scale items

- Line 379: The manuscript reports significant p values such as “less than .000.” This should be revised to “p < .001.”

We corrected the above mistakes.

Good luck with your work!

Sincere thanks to the above three reviewers. Their keen reading of the original manuscript, and thorough suggestions, has led us to revisit the analyses and revise the manuscript in a manner that we hope provides a more clear and compelling case for the value of the study.

Attachment Submitted filename: Response to Reviewers.docx

# Peer review: Decision Letter 1

**DOI:** 10.1371/journal.pone.0359569.r003

**Authors:** Annesha Sil

20 Aug 2026

Dear Dr. Bjornsen,

Thank you for submitting your manuscript to PLOS One. After careful consideration, we feel that it has merit but does not fully meet PLOS One’s publication criteria as it currently stands. Therefore, we invite you to submit a revised version of the manuscript that addresses the points raised during the review process.

Please address the remaining minor concerns raised by the reviewers.

Please submit your revised manuscript by Oct 04 2026 11:59PM. If you will need more time than this to complete your revisions, please reply to this message or contact the journal office at plosone@plos.org. When you're ready to submit your revision, log on to https://www.editorialmanager.com/pone/ and select the 'Submissions Needing Revision' folder to locate your manuscript file.

- A letter that responds to each point raised by the academic editor and reviewer(s). You should upload this letter as a separate file labeled 'Response to Reviewers'.
- A marked-up copy of your manuscript that highlights changes made to the original version. You should upload this as a separate file labeled 'Revised Manuscript with Track Changes'.
- An unmarked version of your revised paper without tracked changes. You should upload this as a separate file labeled 'Manuscript'.

If you would like to make changes to your financial disclosure, please include your updated statement in your cover letter. Guidelines for resubmitting your figure files are available below the reviewer comments at the end of this letter.

If applicable, we recommend that you deposit your laboratory protocols in protocols.io to enhance the reproducibility of your results. Protocols.io assigns your protocol its own identifier (DOI) so that it can be cited independently in the future. For instructions see: https://journals.plos.org/plosone/s/submission-guidelines#loc-laboratory-protocols. Additionally, PLOS One offers an option for publishing peer-reviewed Lab Protocol articles, which describe protocols hosted on protocols.io. Read more information on sharing protocols at https://plos.org/protocols?utm_medium=editorial-email&utm_source=authorletters&utm_campaign=protocols.

As the corresponding author, your ORCID iD is verified in the submission system and will appear in the published article. PLOS supports the use of ORCID, and we encourage all coauthors to register for an ORCID iD and use it as well. Please encourage your coauthors to verify their ORCID iD within the submission system before final acceptance, as unverified ORCID iDs will not appear in the published article. *Only*  the individual author can complete the verification step; PLOS staff *cannot*  verify ORCID iDs on behalf of authors.

We look forward to receiving your revised manuscript.

Kind regards,

Annesha Sil, Ph.D.

Staff Editor

PLOS One

Journal Requirements:

If the reviewer comments include a recommendation to cite specific previously published works, please review and evaluate these publications to determine whether they are relevant and should be cited. There is no requirement to cite these works unless the editor has indicated otherwise.

Please review your reference list to ensure that it is complete and correct. If you have cited papers that have been retracted, please include the rationale for doing so in the manuscript text, or remove these references and replace them with relevant current references. Any changes to the reference list should be mentioned in the rebuttal letter that accompanies your revised manuscript. If you need to cite a retracted article, indicate the article’s retracted status in the References list and also include a citation and full reference for the retraction notice.

[Note: HTML markup is below. Please do not edit.]

Reviewers' comments:

Reviewer's Responses to Questions

**Comments to the Author**

Reviewer #1: All comments have been addressed

Reviewer #2: All comments have been addressed

Reviewer #3: (No Response)

Reviewer #4: All comments have been addressed

**********

2. Is the manuscript technically sound, and do the data support the conclusions?

Reviewer #1: Yes

Reviewer #2: Yes

Reviewer #3: Yes

Reviewer #4: Yes

**********

3. Has the statistical analysis been performed appropriately and rigorously?

Reviewer #1: Yes

Reviewer #2: Yes

Reviewer #3: Yes

Reviewer #4: Yes

**********

4. Have the authors made all data underlying the findings in their manuscript fully available?

The PLOS Data policy

Reviewer #1: Yes

Reviewer #2: Yes

Reviewer #3: Yes

Reviewer #4: Yes

**********

5. Is the manuscript presented in an intelligible fashion and written in standard English?

Reviewer #1: Yes

Reviewer #2: Yes

Reviewer #3: Yes

Reviewer #4: Yes

**********

Reviewer #1: I would like to thank the authors for the effort they have put into addressing the comments made by all the reviewers. I believe the discussion has substantially improved the article and its presentation. As far as I am concerned, I believe the suggested changes have been incorporated, and where they have not been implemented, this has been properly justified. Congratulations.

Reviewer #2: Thank you for having considered by comments and alterations. The manuscript has been considerably improved and the study seems to tell an interesting and comprehensive "story". The introduction has been more focused and alot of issues have been elaborated enhancing coherence and meaning making in terms of the links among the different sections of the study.

Reviewer #3: Dear Author(s), thank you for re-submitting the manuscript Attachment, Grandiose and Vulnerable Narcissism, Interpersonal Competence, and Problematic Social Media Use: A Latent Profile Analysis. The revision is substantially responsive to the initial comments, and the manuscript has improved greatly into a more coherent person-centered paper. The authors now clearly state that the variable-centered analyses are secondary and that the main contribution lies in identifying attachment-related latent profiles and examining their differences in problematic social media use. However, some remaining technical and presentation issues need to be corrected for the paper to be fully publishable. Below you can find recommendations for improvement:

- The authors have substantially improved the treatment of the PSMU measure, describing a split-half validation approach, including EFA in one half, CFA in the other half, CFA in the full sample, KMO, Bartlett’s test, AVE, α, and ω. Although this does not fully solve the lack of external validation, it meaningfully strengthens the manuscript. That said, the authors should still acknowledge that the measure was not externally validated against an established measure such as the Bergen Social Media Addiction Scale or Social Media Disorder Scale. The CFA RMSEA values are also relatively high (.10 in the split-half CFA and .09 in the full-sample CFA), so describing the fit as simply “good” may be too strong. A more cautious phrasing would be preferable.

- The manuscript states that “lower values” indicate better log-likelihood, BIC, SABIC, and AIC. This is correct for AIC/BIC/SABIC, but not for log-likelihood as higher/less negative log-likelihood values indicate better fit. The authors should revise this sentence.

- Check again the sample items for attachment anxiety and attachment avoidance as they seem to be reversed. “I don’t feel comfortable opening up to romantic partners” reflects avoidance, whereas “I worry about being abandoned” reflects anxiety. This should be corrected because Table 1 was added specifically to improve clarity.

- The Discussion is much improved, but the practical implications could be stated more directly: for example, how identifying fearful versus anxious profiles may help researchers, clinicians, or prevention programs better understand risk for problematic social media use.

- Several minor typographical and wording errors need attention, (e.g. “non-signficant,” “the the ECR-12,” “alinged,” “instrusions,” and “participants who with.”)

Good luck with your work!

Reviewer #4: Thank you for the revised version of this paper.

After reading it, I can say that this manuscript has improved clearly on the basis of the p`revious comments provided, and I think the main concerns raised in the previous round have been addressed satisfactorily. The revised analyses are more coherent, the four-profile solution is easier to justify theoretically, and the manuscript now reads much more clearly in terms of its primary objective and overall structure. In my view, the paper is now very close to being acceptable, with only a few minor points that would benefit from a final round of polishing.

My remaining comments are minor and mostly editorial:

Page 14, lines 244-5: please correct “for which there we no missing data on scale items.” This appears to be a simple wording error and should read “were no missing data.”

Page 19, lines 379-80: please revise “the 4-profile solution (a) was the highest profile after which the model became non-significant for the first time…”. The wording is slightly awkward and could be made more direct.

Page 22, lines 420-1: the sentence reporting the post-hoc comparisons is clear, but I would standardize the p-value style throughout the manuscript. In particular, expressions such as “all p ≤ .023” should follow the same formatting conventions used elsewhere in the paper.

Page 24, lines 457-62: the transition into the variable-centered results is still a bit long. Since the person-centered component is now the main contribution, this opening sentence or two could be tightened slightly so the discussion reaches the profile interpretation more quickly.

Page 30, lines 628-42: the conclusions are now much better aligned with the revised profile solution, but I would still suggest one final check to ensure that all references to the earlier six-profile version have been fully removed from the manuscript and tracked-changes file.

Overall, I think the manuscript is in good shape and would support it after these minor adjustments.

Thanks for your contribution.

**********

what does this mean?). If published, this will include your full peer review and any attached files.

If you choose “no”, your identity will remain anonymous but your review may still be made public.

**Do you want your identity to be public for this peer review?**  For information about this choice, including consent withdrawal, please see our Privacy Policy

Reviewer #1: **Yes:**  Alfredo Zarco Alpuente

Reviewer #2: No

Reviewer #3: No

Reviewer #4: No

**********

[NOTE: If reviewer comments were submitted as an attachment file, they will be attached to this email and accessible via the submission site. Please log into your account, locate the manuscript record, and check for the action link "View Attachments". If this link does not appear, there are no attachment files.]

To ensure your figures meet our technical requirements, please review our figure guidelines: https://journals.plos.org/plosone/s/figures

You may also use PLOS’s free figure tool, NAAS, to help you prepare publication quality figures: https://journals.plos.org/plosone/s/figures#loc-tools-for-figure-preparation.

NAAS will assess whether your figures meet our technical requirements by comparing each figure against our figure specifications.

# Author comment: Author response to Decision Letter 2

**DOI:** 10.1371/journal.pone.0359569.r004

25 Aug 2026

We thank the reviewers again for their thorough examination of our revised manuscript. Their suggestions have led us to revise the manuscript in ways that strengthen the results and text, and we hope will benefit readers.

Below are comments and suggestions from Reviewers, followed by our indented replies to each suggestion.

======================================

6. Review Comments to the Author

Reviewer #1: I would like to thank the authors for the effort they have put into addressing the comments made by all the reviewers. I believe the discussion has substantially improved the article and its presentation. As far as I am concerned, I believe the suggested changes have been incorporated, and where they have not been implemented, this has been properly justified. Congratulations.

Reviewer #2: Thank you for having considered by comments and alterations. The manuscript has been considerably improved and the study seems to tell an interesting and comprehensive "story". The introduction has been more focused and alot of issues have been elaborated enhancing coherence and meaning making in terms of the links among the different sections of the study.

We are grateful to Reviewers #1 and #2 for their expertise and very helpful suggestions regarding our work.

Reviewer #3: Dear Author(s), thank you for re-submitting the manuscript Attachment, Grandiose and Vulnerable Narcissism, Interpersonal Competence, and Problematic Social Media Use: A Latent Profile Analysis. The revision is substantially responsive to the initial comments, and the manuscript has improved greatly into a more coherent person-centered paper. The authors now clearly state that the variable-centered analyses are secondary and that the main contribution lies in identifying attachment-related latent profiles and examining their differences in problematic social media use. However, some remaining technical and presentation issues need to be corrected for the paper to be fully publishable.

We are grateful for Reviewer #3's expertise, as well as the suggestions below.

Below you can find recommendations for improvement:

- The authors have substantially improved the treatment of the PSMU measure, describing a split-half validation approach, including EFA in one half, CFA in the other half, CFA in the full sample, KMO, Bartlett’s test, AVE, α, and ω. Although this does not fully solve the lack of external validation, it meaningfully strengthens the manuscript. That said, the authors should still acknowledge that the measure was not externally validated against an established measure such as the Bergen Social Media Addiction Scale or Social Media Disorder Scale.

We have added this acknowledgement in the 'Limitations and future research' section.

The CFA RMSEA values are also relatively high (.10 in the split-half CFA and .09 in the full-sample CFA), so describing the fit as simply “good” may be too strong. A more cautious phrasing would be preferable.

We have revised our manuscript to describe our model fit results as 'acceptable' rather than 'good'.

- The manuscript states that “lower values” indicate better log-likelihood, BIC, SABIC, and AIC. This is correct for AIC/BIC/SABIC, but not for log-likelihood as higher/less negative log-likelihood values indicate better fit. The authors should revise this sentence.

We have corrected the text in the Statistical analyses section and the Results section.

- Check again the sample items for attachment anxiety and attachment avoidance as they seem to be reversed. “I don’t feel comfortable opening up to romantic partners” reflects avoidance, whereas “I worry about being abandoned” reflects anxiety. This should be corrected because Table 1 was added specifically to improve clarity.

Thank you kindly. We have corrected this error in Table 1.

- The Discussion is much improved, but the practical implications could be stated more directly: for example, how identifying fearful versus anxious profiles may help researchers, clinicians, or prevention programs better understand risk for problematic social media use.

We have added a second-to-last paragraph in the discussion addressing the practical implications as noted above.

- Several minor typographical and wording errors need attention, (e.g. “non-signficant,” “the the ECR-12,” “alinged,” “instrusions,” and “participants who with.”)

Good luck with your work!

Thank you kindly. We have corrected the errors noted above.

Reviewer #4: Thank you for the revised version of this paper.

After reading it, I can say that this manuscript has improved clearly on the basis of the p`revious comments provided, and I think the main concerns raised in the previous round have been addressed satisfactorily. The revised analyses are more coherent, the four-profile solution is easier to justify theoretically, and the manuscript now reads much more clearly in terms of its primary objective and overall structure. In my view, the paper is now very close to being acceptable, with only a few minor points that would benefit from a final round of polishing.

My remaining comments are minor and mostly editorial:

Page 14, lines 244-5: please correct “for which there we no missing data on scale items.” This appears to be a simple wording error and should read “were no missing data.”

We corrected this error.

Page 19, lines 379-80: please revise “the 4-profile solution (a) was the highest profile after which the model became non-significant for the first time…”. The wording is slightly awkward and could be made more direct.

We edited this text as noted.

Page 22, lines 420-1: the sentence reporting the post-hoc comparisons is clear, but I would standardize the p-value style throughout the manuscript. In particular, expressions such as “all p ≤ .023” should follow the same formatting conventions used elsewhere in the paper.

We removed “all p ≤ .023” and point the reader to specific comparisons and p values in Table 4, rather than reiterate all six sets of results in the paragraph text.

Page 24, lines 457-62: the transition into the variable-centered results is still a bit long. Since the person-centered component is now the main contribution, this opening sentence or two could be tightened slightly so the discussion reaches the profile interpretation more quickly.

We edited the text to eliminate unnecessary wording.

Page 30, lines 628-42: the conclusions are now much better aligned with the revised profile solution, but I would still suggest one final check to ensure that all references to the earlier six-profile version have been fully removed from the manuscript and tracked-changes file.

We have carefully reviewed the Discussion section as suggested.

Overall, I think the manuscript is in good shape and would support it after these minor adjustments.

Thanks for your contribution.

Our very sincere thanks to all reviewers.

Attachment Submitted filename: Response_to_Reviewers_auresp_2.docx

# Peer review: Decision Letter 2

**DOI:** 10.1371/journal.pone.0359569.r005

**Authors:** Srebrenka Letina

8 Sep 2026

Dear Dr. Bjornsen,

Thank you for submitting your manuscript to PLOS One. After careful consideration, we feel that it has merit but does not fully meet PLOS One’s publication criteria as it currently stands. Therefore, we invite you to submit a revised version of the manuscript that addresses the points raised during the review process.

Again, two reviewers now recommend acceptance, while the third reviewer finds that the manuscript has improved substantially and raises only a small number of remaining issues.

Please address the remaining comments, particularly by qualifying the interpretation of the CFA model fit, avoiding causal or developmental claims that are not supported by the cross-sectional design, streamlining the opening of the Discussion, clarifying the wording in Table 3, and conducting a final careful proofread.

I therefore invite you to submit a minor revision addressing these points. Provided the remaining issues are adequately addressed, the next evaluation will be handled editorially and the manuscript will not be sent back to the three reviewers.

Please submit your revised manuscript by Oct 23 2026 11:59PM. If you will need more time than this to complete your revisions, please reply to this message or contact the journal office at plosone@plos.org. When you're ready to submit your revision, log on to https://www.editorialmanager.com/pone/ and select the 'Submissions Needing Revision' folder to locate your manuscript file.

- A letter that responds to each point raised by the academic editor and reviewer(s). You should upload this letter as a separate file labeled 'Response to Reviewers'.
- A marked-up copy of your manuscript that highlights changes made to the original version. You should upload this as a separate file labeled 'Revised Manuscript with Track Changes'.
- An unmarked version of your revised paper without tracked changes. You should upload this as a separate file labeled 'Manuscript'.

If you would like to make changes to your financial disclosure, please include your updated statement in your cover letter. Guidelines for resubmitting your figure files are available below the reviewer comments at the end of this letter.

If applicable, we recommend that you deposit your laboratory protocols in protocols.io to enhance the reproducibility of your results. Protocols.io assigns your protocol its own identifier (DOI) so that it can be cited independently in the future. For instructions see: https://journals.plos.org/plosone/s/submission-guidelines#loc-laboratory-protocols. Additionally, PLOS One offers an option for publishing peer-reviewed Lab Protocol articles, which describe protocols hosted on protocols.io. Read more information on sharing protocols at https://plos.org/protocols?utm_medium=editorial-email&utm_source=authorletters&utm_campaign=protocols.

As the corresponding author, your ORCID iD is verified in the submission system and will appear in the published article. PLOS supports the use of ORCID, and we encourage all coauthors to register for an ORCID iD and use it as well. Please encourage your coauthors to verify their ORCID iD within the submission system before final acceptance, as unverified ORCID iDs will not appear in the published article. *Only*  the individual author can complete the verification step; PLOS staff *cannot*  verify ORCID iDs on behalf of authors.

We look forward to receiving your revised manuscript.

Kind regards,

Srebrenka Letina, Ph.D.

Academic Editor

PLOS One

Journal Requirements:

1. If the reviewer comments include a recommendation to cite specific previously published works, please review and evaluate these publications to determine whether they are relevant and should be cited. There is no requirement to cite these works unless the editor has indicated otherwise.

2. Please review your reference list to ensure that it is complete and correct. If you have cited papers that have been retracted, please include the rationale for doing so in the manuscript text, or remove these references and replace them with relevant current references. Any changes to the reference list should be mentioned in the rebuttal letter that accompanies your revised manuscript. If you need to cite a retracted article, indicate the article’s retracted status in the References list and also include a citation and full reference for the retraction notice.

[Note: HTML markup is below. Please do not edit.]

Reviewers' comments:

Reviewer's Responses to Questions

**Comments to the Author**

Reviewer #1: All comments have been addressed

Reviewer #2: All comments have been addressed

Reviewer #3: (No Response)

**********

2. Is the manuscript technically sound, and do the data support the conclusions?

Reviewer #1: Yes

Reviewer #2: Yes

Reviewer #3: Yes

**********

3. Has the statistical analysis been performed appropriately and rigorously?

Reviewer #1: Yes

Reviewer #2: Yes

Reviewer #3: Yes

**********

4. Have the authors made all data underlying the findings in their manuscript fully available?

The PLOS Data policy

Reviewer #1: Yes

Reviewer #2: Yes

Reviewer #3: Yes

**********

5. Is the manuscript presented in an intelligible fashion and written in standard English?

Reviewer #1: Yes

Reviewer #2: Yes

Reviewer #3: Yes

**********

Reviewer #1: I had already considered the manuscript suitable for publication in the previous round. Having reviewed the suggestions from the other reviewers and the authors’ corresponding revisions, I maintain my positive assessment and consider the manuscript suitable for publication.

Reviewer #2: The paper has been considerably improved and the suggestions and alterations have been taken into account. From my perspective, it is at a stage that can be published.

Reviewer #3: Dear Author(s), thank you for the careful revision. The manuscript has improved substantially, and the authors have adequately addressed most of the substantive concerns raised in the previous round. The four-profile solution is now presented more clearly, the log-likelihood interpretation and attachment sample items have been corrected, the absence of external validation of the PSMU measure is acknowledged, and references to the earlier six-profile solution appear to have been removed.

A small number of issues remain before the manuscript is ready for acceptance:

1. On p. 15, lines 287–291, the CFA results are described as demonstrating “overall acceptable model fit.” Because RMSEA was .10 in the holdout sample and .09 in the full sample—both above the authors’ stated criterion of ≤ .08—the results would be more accurately described as showing mixed fit: acceptable according to CFI, TLI, and SRMR, but weaker fit according to RMSEA.

2. The practical-implications paragraph on pp. 29–30, lines 586–593 addresses the previous revision’s comment, but its wording should be revisited. In particular, statements that avoidant attachment “does not predict” high PSMU and that individuals are “at greater risk of failing to develop necessary interpersonal skills” imply causal or developmental conclusions that cannot be supported by this cross-sectional study. The sentence beginning “Yet, of practical use to researchers…” also requires grammatical revision.

3. The opening of the Discussion remains somewhat repetitive. The sentences “We interpret these findings in greater detail below” and “Below we briefly interpret…” could be combined so that the discussion moves more directly to the findings. Also, I suggest that you omit the reference to the H8 and H9 hypothesis at the beginning of the chapter, as it reads confusing. Leave only the sentence without the two brackets. You can mention them in the right order afterwards.

4. A final proofread is required. Remaining errors include, for example, “alinged” (p. 21, line 404), “naricissistic” (p. 27, line 523), “signficant” (p. 32, line 640), “handing” rather than “handling” in Table 1.

5. In Table 3, “Bold indicates best fitted model” would be more accurate as “Bold indicates the selected model,” because different indices favour different solutions and the four-profile model was selected through a combination of statistical and theoretical considerations.

After these minor corrections I believe the manuscript could be suitable for publication.

**********

what does this mean?). If published, this will include your full peer review and any attached files.

If you choose “no”, your identity will remain anonymous but your review may still be made public.

**Do you want your identity to be public for this peer review?**  For information about this choice, including consent withdrawal, please see our Privacy Policy

Reviewer #1: No

Reviewer #2: No

Reviewer #3: No

**********

[NOTE: If reviewer comments were submitted as an attachment file, they will be attached to this email and accessible via the submission site. Please log into your account, locate the manuscript record, and check for the action link "View Attachments". If this link does not appear, there are no attachment files.]

To ensure your figures meet our technical requirements, please review our figure guidelines: https://journals.plos.org/plosone/s/figures

You may also use PLOS’s free figure tool, NAAS, to help you prepare publication quality figures: https://journals.plos.org/plosone/s/figures#loc-tools-for-figure-preparation.

NAAS will assess whether your figures meet our technical requirements by comparing each figure against our figure specifications.

# Author comment: Author response to Decision Letter 3

**DOI:** 10.1371/journal.pone.0359569.r006

14 Sep 2026

We thank the reviewers again for their thorough examination of our revised manuscript. Their suggestions have led us to further revise and improve the manuscript, which we hope will benefit readers.

Below are comments and suggestions from Reviewers, followed by our indented replies to each suggestion.

======================================

Reviewer #3: Dear Author(s), thank you for the careful revision. The manuscript has improved substantially, and the authors have adequately addressed most of the substantive concerns raised in the previous round. The four-profile solution is now presented more clearly, the log-likelihood interpretation and attachment sample items have been corrected, the absence of external validation of the PSMU measure is acknowledged, and references to the earlier six-profile solution appear to have been removed.

A small number of issues remain before the manuscript is ready for acceptance:

1. On p. 15, lines 287–291, the CFA results are described as demonstrating “overall acceptable model fit.” Because RMSEA was .10 in the holdout sample and .09 in the full sample—both above the authors’ stated criterion of ≤ .08—the results would be more accurately described as showing mixed fit: acceptable according to CFI, TLI, and SRMR, but weaker fit according to RMSEA.

Thank you. We have made the above change to the text on page 15.

2. The practical-implications paragraph on pp. 29–30, lines 586–593 addresses the previous revision’s comment, but its wording should be revisited. In particular, statements that avoidant attachment “does not predict” high PSMU and that individuals are “at greater risk of failing to develop necessary interpersonal skills” imply causal or developmental conclusions that cannot be supported by this cross-sectional study.

Thank you. We have edited the language on pages 29-30 to remove any implication of causal or developmental conclusions. We also noticed and edited text in the first Discussion paragraph (p. 23) for the same purpose (e.g., we replaced "develop" with "express" on line 454, Track Changes MS).

The sentence beginning “Yet, of practical use to researchers…” also requires grammatical revision.

Thank you. We have edited the referenced sentence.

3. The opening of the Discussion remains somewhat repetitive. The sentences “We interpret these findings in greater detail below” and “Below we briefly interpret…” could be combined so that the discussion moves more directly to the findings. Also, I suggest that you omit the reference to the H8 and H9 hypothesis at the beginning of the chapter, as it reads confusing. Leave only the sentence without the two brackets. You can mention them in the right order afterwards.

Thank you. We have made the above corrections.

4. A final proofread is required. Remaining errors include, for example, “alinged” (p. 21, line 404), “naricissistic” (p. 27, line 523), “signficant” (p. 32, line 640), “handing” rather than “handling” in Table 1.

Thank you. We have made the above corrections. We also noticed and corrected a grammatical error in the Abstract (added 'the' on line 40), and a wording error in the section Limitations and future research (line 627 Track Changes MS, changed 'Fourth' to 'Third').

5. In Table 3, “Bold indicates best fitted model” would be more accurate as “Bold indicates the selected model,” because different indices favour different solutions and the four-profile model was selected through a combination of statistical and theoretical considerations.

Thank you. We have made the above corrections.

After these minor corrections I believe the manuscript could be suitable for publication.

We are sincerely grateful to all reviewers for their thorough and expert reviews of our manuscript.

Attachment Submitted filename: Response_to_Reviewers_auresp_3.docx

# Editor report: Decision Letter 3

**DOI:** 10.1371/journal.pone.0359569.r007

**Authors:** Srebrenka Letina

15 Sep 2026

Attachment, grandiose and vulnerable narcissism, interpersonal competence, and problematic social media use: A latent profile analysis.

PONE-D-25-44345R3

Dear Dr. Bjornsen,

We’re pleased to inform you that your manuscript has been judged scientifically suitable for publication and will be formally accepted for publication once it meets all outstanding technical requirements.

Within one week, you’ll receive an e-mail detailing the required amendments. When these have been addressed, you’ll receive a formal acceptance letter and your manuscript will be scheduled for publication.

An invoice will be generated when your article is formally accepted. Please note, if your institution has a publishing partnership with PLOS and your article meets the relevant criteria, all or part of your publication costs will be covered. Please make sure your user information is up-to-date by logging into Editorial Manager at Editorial Manager® and clicking the ‘Update My Information' link at the top of the page. For questions related to billing, please contact billing support.

If your institution or institutions have a press office, please notify them about your upcoming paper to help maximize its impact. If they’ll be preparing press materials, please inform our press team as soon as possible -- no later than 48 hours after receiving the formal acceptance. Your manuscript will remain under strict press embargo until 2 pm Eastern Time on the date of publication. For more information, please contact onepress@plos.org.

Kind regards,

Srebrenka Letina, Ph.D.

Academic Editor

PLOS One

Additional Editor Comments (optional):

Reviewers' comments:

# Editor report: Acceptance letter

**DOI:** 10.1371/journal.pone.0359569.r008

**Authors:** Srebrenka Letina

PONE-D-25-44345R3

PLOS One

Dear Dr. Bjornsen,

I'm pleased to inform you that your manuscript has been deemed suitable for publication in PLOS One. Congratulations! Your manuscript is now being handed over to our production team.

At this stage, our production department will prepare your paper for publication. This includes ensuring the following:

* All references, tables, and figures are properly cited

* All relevant supporting information is included in the manuscript submission,

* There are no issues that prevent the paper from being properly typeset

You will receive further instructions from the production team, including instructions on how to review your proof when it is ready. Please keep in mind that we are working through a large volume of accepted articles, so please give us a few days to review your paper and let you know the next and final steps.

Lastly, if your institution or institutions have a press office, please let them know about your upcoming paper now to help maximize its impact. If they'll be preparing press materials, please inform our press team within the next 48 hours. Your manuscript will remain under strict press embargo until 2 pm Eastern Time on the date of publication. For more information, please contact onepress@plos.org.

You will receive an invoice from PLOS for your publication fee after your manuscript has reached the completed accept phase. If you receive an email requesting payment before acceptance or for any other service, this may be a phishing scheme. Learn how to identify phishing emails and protect your accounts at https://explore.plos.org/phishing.

If we can help with anything else, please email us at customercare@plos.org.

Thank you for submitting your work to PLOS One and supporting open access.

Kind regards,

PLOS One Editorial Office Staff

on behalf of

Dr. Srebrenka Letina

Academic Editor

PLOS One
